# Recently Integrated *Alu* Elements in Capuchin Monkeys: A Resource for *Cebus*/*Sapajus* Genomics

**DOI:** 10.3390/genes13040572

**Published:** 2022-03-24

**Authors:** Jessica M. Storer, Jerilyn A. Walker, Catherine E. Rockwell, Grayce Mores, Thomas O. Beckstrom, Joseph D. Orkin, Amanda D. Melin, Kimberley A. Phillips, Christian Roos, Mark A. Batzer

**Affiliations:** 1 Department of Biological Sciences, Louisiana State University, 202 Life Sciences Building, Baton Rouge, LA 70803, USA; jessica.storer1024@gmail.com (J.M.S.); jawalker@lsu.edu (J.A.W.); crockw1@lsu.edu (C.E.R.); gmores1@lsu.edu (G.M.); tbecks@uw.edu (T.O.B.); 2 Institute for Systems Biology, Seattle, WA 98109, USA; 3 Department of Oral and Maxillofacial Surgery, University of Washington, 1959 NE Pacific Street, Health Sciences Building B-241, Seattle, WA 98195, USA; 4 Département d’Anthropologie, Université de Montréal, 3150 Jean-Brillant, Montréal, QC H3T 1N8, Canada; joseph.orkin@upf.edu; 5 Institute of Evolutionary Biology (UPF-CSIC), PRBB, Dr. Aiguader 88, 08003 Barcelona, Spain; 6 Department of Anthropology and Archaeology & Department of Medical Genetics, University of Calgary, 2500 University Drive, N.W., Calgary, AB T2N 1N4, Canada; amanda.melin@ucalgary.ca; 7 Alberta Children’s Hospital Research Institute, Heritage Medical Research Building, 3330 Hospital Drive, N.W., Calgary, AB T2N 4N1, Canada; 8 Department of Psychology, Trinity University, 1 Trinity Place, San Antonio, TX 78212, USA; kimberley.phillips@trinity.edu; 9 Southwest National Primate Research Center, Texas Biomedical Research Institute, San Antonio, TX 78245, USA; 10 Gene Bank of Primates and Primate Genetics Laboratory, German Primate Center, Leibniz Institute for Primate Research, 37077 Göttingen, Germany; croos@dpz.eu

**Keywords:** Cebidae, capuchin, *Alu*, *Cebus*, *Sapajus*, phylogeny, platyrrhine

## Abstract

Capuchins are platyrrhines (monkeys found in the Americas) within the Cebidae family. For most of their taxonomic history, the two main morphological types of capuchins, gracile (untufted) and robust (tufted), were assigned to a single genus, *Cebus*. Further, all tufted capuchins were assigned to a single species, *Cebus apella*, despite broad geographic ranges spanning Central and northern South America. In 2012, tufted capuchins were assigned to their genus, *Sapajus*, with eight currently recognized species and five *Cebus* species, although these numbers are still under debate. *Alu* retrotransposons are a class of mobile element insertion (MEI) widely used to study primate phylogenetics. However, *Alu* elements have rarely been used to study capuchins. Recent genome-level assemblies for capuchins (*Cebus imitator*; [Cebus_imitator_1.0] and *Sapajus apella* [GSC_monkey_1.0]) facilitated large scale ascertainment of young lineage-specific *Alu* insertions. Reported here are 1607 capuchin specific and 678 *Sapajus* specific *Alu* insertions along with candidate oligonucleotides for locus-specific PCR assays for many elements. PCR analyses identified 104 genus level and 51 species level *Alu* insertion polymorphisms. The *Alu* datasets reported in this study provide a valuable resource that will assist in the classification of archival samples lacking phenotypic data and for the study of capuchin phylogenetic relationships.

## 1. Introduction

Capuchins constitute a monophyletic clade of platyrrhines (monkeys found in the Americas) within the Cebidae family, having an estimated origin of about 6.8 million years ago (mya) [1,2]. Capuchins diverged from their most closely related sister taxon within Cebidae, the squirrel monkeys (genus *Saimiri*) approximately 13.8 mya [1]. Recognized in popular folklore as the ‘organ grinder’ monkey or Cebus monkey, capuchins comprise a diverse group of morphologically and phenotypically distinct members, distributed across a broad geographic range of Central and northern South America. The current consensus of capuchin systematics is that they are represented by two genera, the *Cebus* genus, with at least five species, *Cebus albifrons*, *Cebus capucinus, Cebus imitator, Cebus olivaceus* and *Cebus kaapori* [2,3,4] and the *Sapajus* genus, with eight recognized species, *Sapajus xanthosternos*, *Sapajus nigritus*, *Sapajus robustus*, *Sapajus flavius*, *Sapajus libidinosus*, *Sapajus cay*, *Sapajus apella,* and *Sapajus macrocephalus* [2,5]. The geographic distribution of capuchins across maps of South and Central America is nicely illustrated in Lynch Alfaro et al. [6] and Martins-Junior et al. [2].

However, the taxonomy of capuchin monkeys has traditionally not been this refined. Historically, it has been confusing due to changing nomenclature as well as periodic reassessments of the number of species and subspecies [3,7,8]. Early taxonomists grouped all capuchins into a single genus, *Cebus*, even though morphological differences suggested two distinct types [5] that were later categorized as tufted and untufted, or robust and gracile, respectively [1]. Further, the name *Cebus apella* was used to encompass all tufted (robust) capuchins into a single species, a nomenclature that remained widely used until the recent decade. Alfaro et al. 2012 [1] proposed the use of *Sapajus* for tufted (brown; robust) capuchins and suggested that the genus *Cebus* be restricted to only the untufted (gracile) capuchins. A roundtable of capuchin researchers held at the International Primatological Society Congress in 2012 adopted the use of the term *Sapajus* as an urgent research priority, calling for the immediate end of the name *Cebus apella* [9].

**Figure 1:**
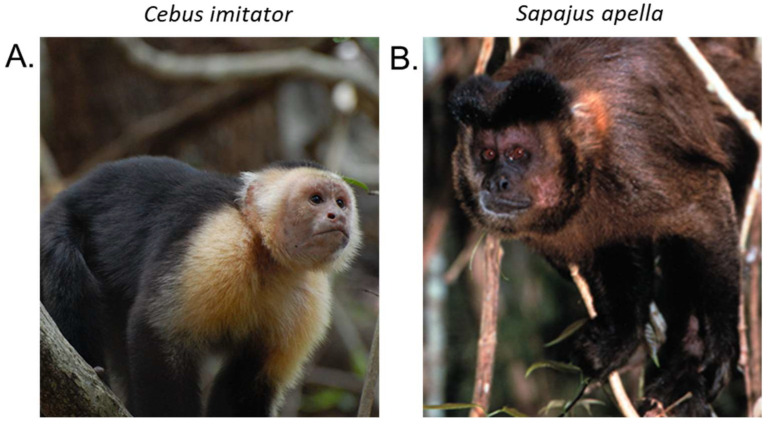
Photographs representative of untufted and tufted capuchins. (**A**) *C. imitator*, an untufted, adult male Panamanian white-faced capuchin. Photograph by Amanda Melin. (**B**) *S. apella*, a tufted, brown capuchin [14].

Photographic images of tufted and untufted capuchins are shown in Figure 1. Additional illustrations of variations in facial phenotypes among several capuchin species are available in Alfaro et al. [1]. Despite obvious phenotypic distinction between the two general forms, and widespread diversity among various species, the confusing historical nomenclature has created a situation in which most archival tissue and DNA samples for the brown tufted capuchins are labeled as *Cebus apella* or just Cebus monkey. Those for untufted gracile capuchins are also labeled Cebus monkey. This makes it difficult to determine the actual taxonomic origin when phenotypic descriptions are unavailable. In addition, some species in both genera are considered Endangered such as *S. robustus* [10], or Critically Endangered such as *C. kaapori* [11], making it essential to have reliable taxonomic, genetic markers for effective conservation efforts. A recent study conducted on populations of *S. libidinosus*, a species considered “Near Threatened” [12], emphasized that there is currently a lack of developed genetic systems available to study capuchin population and conservation genetics [13].

Primate specific *Alu* retrotransposons are well-established diagnostic genetic markers for the study of population genetic and phylogenetic relationships [15,16,17,18,19,20,21,22,23,24,25,26,27,28,29,30,31]. Non-autonomous *Alu* elements mobilize via a “copy and paste” mechanism through an RNA intermediate, utilizing the enzymatic machinery of autonomous LINE (L1) elements. This mode of mobilization is termed “target-primed reverse transcription” (TPRT) [32]. The TPRT integration process produces 5′ and 3′ flanking target site duplications (TSDs) that can be used to identify each insertion. TPRT is considered a unidirectional mode of integration such that the ancestral state is considered the absence of an insertion, and shared insertions with matching TSDs are accepted as being inherited from a common ancestor. *Alu* subfamilies evolve by the stepwise accumulation of diagnostic nucleotide arrangements such that each primate lineage derives a unique group of *Alu* subfamilies [33,34,35,36,37,38]. The oldest subfamily, *Alu*J, is found in all primates, whereas *Alu*S was active after the separation of Strepsirrhini and Tarsiiformes from Platyrrhini and Catarrhini [36,37], and subfamily *Alu*Y is found only in catarrhines [33]. Platyrrhine-specific *Alu* element subfamilies include *Alu*Ta7, *Alu*Ta10, and *Alu*Ta15, with Ta15 thought to be limited to the Cebidae family [21]. More recently, other platyrrhine *Alu* subfamilies have been characterized in marmoset [39], squirrel monkey [40], capuchin, and owl monkeys [41,42]. Previous studies have utilized *Alu* elements in platyrrhine phylogeny [15,31,43,44,45], and although capuchin monkey studies have been conducted using *Alu* insertions, they involved a relatively small number of elements [44,46]. Recently, genome-level assemblies have become available for capuchins (*C. imitator*; [Cebus_imitator-1.0] and *S. apella* [GSC_monkey_1.0]). This study aimed to computationally ascertain a dataset of the youngest *Alu* insertions specific to these capuchin lineages and to perform locus-specific PCR on a subset of each to identify *Alu* insertion polymorphisms. The *Alu* datasets reported in this study provide a valuable resource that will assist in the classification of archival samples and facilitate future studies of capuchin phylogeny, populations genetics, and conservation strategies.

## 2. Materials and Methods

### 2.1. Lineage-Specific Alu Elements

Ascertainment of lineage-specific *Alu* insertions from the *C. imitator* genome [Cebus_imitator-1.0] [47] was performed as previously described [41,42]. Briefly, the [Cebus_imitator-1.0] genome was obtained from the National Center for Biotechnology Information (NCBI) and analyzed for full-length *Alu* elements with RepeatMasker [48] (RepeatMasker-Open-4.0). Full-length *Alu* elements are described as having a start site within 4 bp of its consensus sequence and being 267 bp or longer. A sequential BLAT [49] of the full-length *Alu* elements extracted from RepeatMasker was conducted with human (*Homo sapiens*; GRCh38.p13), common marmoset (*Callithrix jacchus*; caljac3), owl monkey (*Aotus nancymaae*; Anan_2.0), and squirrel monkey (*Saimiri boliviensis*; SaiBol1.0) genomes in that order. A sequential BLAT involved analyzing the output after each BLAT for capuchin-specific *Alu* elements compared to the other four genomes. The genome assembly for *S. apella*, [GSC_monkey_1.0] was not available at that time. Capuchin lineage-specific *Alu* insertions were aligned using Crossmatch (www.phrap.org/phredphrapconsed.html; accessed on 4 March 2022), and *Alu* subfamily structure was determined using COSEG (www.repeatmasker.org/COSEGDownload.html; accessed on 4 March 2022).

The percent divergence of the lineage-specific *Alu* elements compared to their respective consensus sequences was determined using an in-house installation of RepeatMasker. Young elements, defined as having less than or equal to two percent sequence divergence, were retained. A custom Python script extracted the FASTA files of the young, lineage-specific elements and sorted them into subfamilies. Five elements for each identified *Alu* subfamily were randomly selected for wet-bench experimental validation. If an *Alu* subfamily had less than five elements, then all lineage-specific *Alu* elements for that subfamily were selected for experimental validation. The orthologous sequence and 600 bp of flanking sequences for human, marmoset, squirrel monkey, and owl monkey genomes were obtained for each *Alu* element using BLAT. BioEdit was used to align the four sequences for each locus [50].

The genome assembly for *S. apella*, [GSC_monkey_1.0], became available more recently, and therefore, ascertainment of the lineage-specific *Alu* insertions from this genome was conducted under consideration of the existing *C. imitator* genome. Full-length *Alu* elements from the *Sapajus* genome were first filtered against the *C. imitator* genome using BLAT, greatly reducing the number of candidates. The output was then filtered using a custom Python script (available on link https://github.com/t-beck; accessed on 4 March 2022) to BLAT the genomes of human, *C. imitator*, marmoset, owl monkey, and squirrel monkey in a single program. These putatively *Sapajus* lineage-specific *Alu* elements, plus 600 bp flanking sequence, were then analyzed using an in-house RepeatMasker library and filtered by position near the center of the FASTA sequence (i.e., 600 bp) to represent the target insertion. Young insertions (≤2% divergence) were retained.

### 2.2. Oligonucleotide Primer Design

Oligonucleotide primers for the polymerase chain reaction (PCR) for *Cebus Alu* elements were designed using Primer3 software [51] with the following modifications: Tm range = 57–63, Max Tm difference = 2, max poly x = 3, min GC content = 40. NCBI Primer Blast [52] was used to analyze the primers for *Cebus* specificity and verify the predicted PCR fragment lengths for *Cebus* and outgroup genomes. For *Sapajus* lineage-specific *Alu* elements, oligonucleotide primers for PCR were designed using an in-house primer design pipeline consisting of a series of custom Python scripts in conjunction with Primer3 (available on link https://github.com/t-beck; accessed on 4 March 2022), followed by screening using NCBI Primer Blast [47]. The oligonucleotide primers were obtained from Sigma Aldrich (Woodlands, TX, USA).

### 2.3. DNA Samples

DNA samples and their origins are described in Supplementary Files S1 and S2. DNA from tissue samples was prepared as described previously [15]. There were two DNA sample panels used in this study, the capuchin monkey panel and the *Sapajus* panel. The capuchin monkey panel included DNA from 14 different capuchin monkeys, six individuals from genus *Cebus,* including the *C. imitator* (sample Cc_AM_T3) used in the reference genome, and eight *S. apella* individuals, all obtained with the original designation ‘*Cebus apella*’ (Supplementary File S1). The *Sapajus* DNA panel included three additional *S. apella* individuals (Supplementary File S2), who were originally labeled as ‘Cebus monkeys’ when acquired initially and were only recently determined to be brown tufted capuchins [53,54].

### 2.4. PCR Amplification

Each DNA sample panel for PCR included a negative control (TLE: 10 mM Tris/0.1 mM EDTA) and four outgroup controls, human (HeLa), the common marmoset (*Callithrix jacchus)*, squirrel monkey (*Saimiri sciureus*), and owl monkey (*Aotus trivirgatus*) representing the pre-integration site, or *Alu* absent PCR amplicon. PCR amplifications were performed in 25 μL reactions containing 25 ng of template DNA, 200 nM of each oligonucleotide primer, 1.5 mM MgCl_2_, 10 × PCR buffer (1×:50 mM KCl; 10 mM TrisHCl, pH 8.4), 0.2 mM dNTPs, and 1–2 U *Taq* DNA polymerase. PCR reactions were performed under the following conditions: initial denaturation at 94 °C for 60 s, followed by 32 cycles of denaturation at 94 °C for 30 s, 30 s at 57 °C annealing temperature, and extension at 72 °C for 30 s. PCRs were completed with a final extension at 72 °C for 2 min. 20 μL of each PCR product were fractionated by size in a horizontal gel chamber on a 2% agarose gel containing 0.2 μg/mL ethidium bromide for 60 minutes at 175 V. UV-fluorescence was used to visualize the DNA fragments, and images were saved using a BioRad ChemiDoc XRS imaging system (Hercules, CA, USA). If PCR results were weak or unresolved, the PCR reaction was repeated using a hot-start with the JumpStart *Taq* DNA polymerase kit (Sigma Aldrich, Woodlands, TX, USA). Following gel electrophoresis, genotypes were recorded in an Excel spreadsheet as (1, 1) for homozygous present, (0, 0) homozygous absent, or (1, 0) heterozygous. “Missing data” was coded as (−9, −9) (Supplementary Files S1 and S2; “Genotypes” worksheet).

### 2.5. Sanger Chain Termination DNA Sequencing

We used traditional Sanger DNA sequencing [55] to resolve certain cases of ambiguity that arose during PCR analyses. Sequence analysis is the best way to avoid erroneous interpretation of PCR patterns. In one *Cebus* locus, some *Alu* present PCR amplicons occurred among *S. apella* samples for an *Alu* absent from the non-reference genome *S. apella*, [GSC_monkey_1.0]. This was sequenced to confirm a shared *Alu* insertion. In four *Sapajus* loci, an *Alu* present PCR amplicon was obtained in a *Cebus* sample, although the *Alu* was absent from that corresponding non-reference genome [Cebus_imitator-1.0]. These were analyzed by Sanger DNA sequencing to confirm a shared insertion or to identify a different *Alu* element in the same region (a near-parallel insertion). If an ambiguous PCR amplicon involved a *C. albifrons* DNA sample, then the *Cebus albifrons* (white-fronted capuchin) (GCA_004027755.1) genome assembly [CebAlb_V1_BIUU] was analyzed for comparison. Sanger DNA sequencing was performed as described previously [56]. Briefly, four PCR fragments per locus were gel purified using a Wizard SV gel purification kit (Promega Corporation, Madison, WI, catalog A9282), 4 µL was used for chain termination cycle sequencing using BigDye Terminator v3.1. Four separate reactions were conducted for each locus using forward or reverse PCR primer; internal-*Alu* primer IntF1: 5′ GGTGGCTCACGCCTGTAATC 3′ [56] and SIntR1: 5′ TCTCGGCTCACCGCAACCTCC 3′ [15]. Following capillary electrophoresis, sequence quality was evaluated using ABI software Sequence Scanner v2.0. Sequence alignments were constructed in BioEdit [50].

## 3. Results

**Figure 2:**
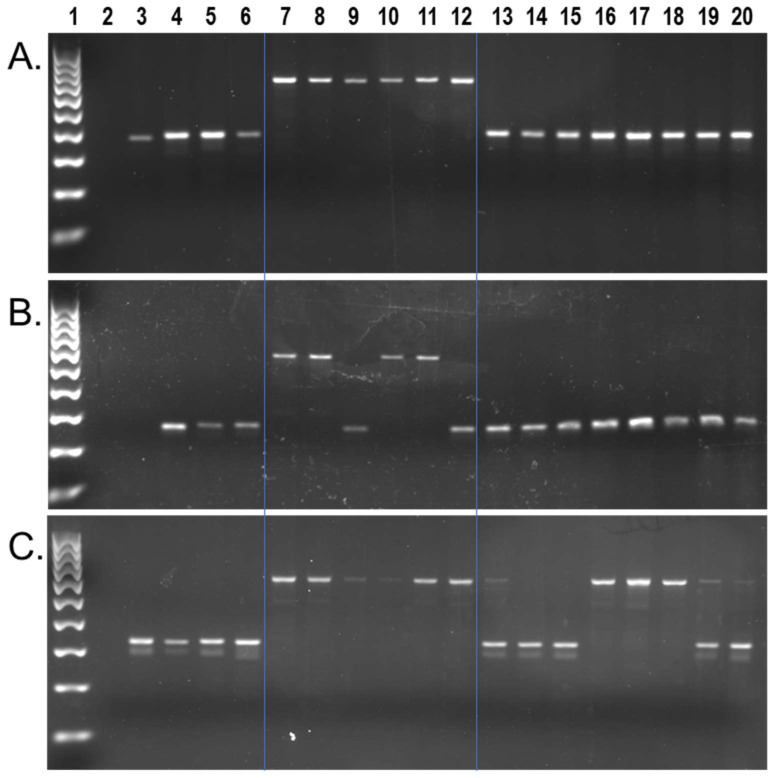
*C. imitator* genomic *Alu* insertion polymorphisms. Lanes: 1 100 bp DNA ladder, 2 TLE (negative control), 3 Human (HeLa), 4 *Callithrix jacchus* (Common marmoset), 5 *Saimiri s. sciureus* (Common squirrel monkey), 6 *Aotus trivirgatus* (Three striped owl monkey), 7 *C. imitator*, 8–9 *C. capucinus*, 10–11 *C. albifrons*, 12 *C. a. albifrons*, 13–20 *S. apella*. (**A**) Locus #69, *Alu* is present in all *Cebus* species individuals (~693 bp DNA fragment lanes 7–12) and absent in all *Sapajus* samples (~381 bp DNA fragment lanes 13–20); (**B**) Locus #49, *Alu* is polymorphic among *Cebus* individuals (~580 bp fragment present, ~275 bp fragment absent) and absent in all *Sapajus* samples; (**C**) Locus #9, *Alu* is present in *Cebus* individuals (~653 bp fragment) and polymorphic among *Sapajus* individuals (~653 bp, and ~340 bp DNA fragments). Blue lines superimposed on gel images visually separate *Cebus*, *Sapajus,* and outgroups.

We found approximately 9602 capuchin lineage-specific *Alu* insertions in the [Cebus_imitator-1.0] genome, from a total of 617,132 full-length insertions [42]. We identified 1607 of these as young (≤2% sequence divergence from their consensus). Local RepeatMasker [48] (RepeatMasker-Open-4.0) output for these loci, along with their genome coordinates and *Alu* subfamily designation are shown in Supplementary File S1. Wet bench locus-specific PCR analyses for a subset of n = 132 young insertions, representing 29 different *Alu* subfamilies, identified n = 84 *Alu* elements that are polymorphic among capuchins on our DNA sample panel. There were n = 54 that were homozygous present in all *Cebus* species individuals while being absent from *Sapajus* samples (Figure 2a), n = 29 that were polymorphic for insertion presence/absence within *Cebus* species samples from *C. imitator*, *C. capucinus* and *C. albifrons*, while absent from *Sapajus* samples (Figure 2b), and n = 1 insertion found to be polymorphic among eight *Sapajus* individuals while being homozygous present in all *Cebus* species individuals (Figure 2c). PCR primer sequences, their predicted amplicon sizes, and the resulting genotypes are available in Supplementary File S1.

To ensure that the locus shown in Figure 2c represented the same *Cebus*-ascertained locus and not a near-parallel insertion, we first constructed a sequence alignment using homologous regions from the *S. apella* genome [GSC_monkey_1.0] and the squirrel monkey genome, genus *Saimiri*, [SaiBol1.0], both of which aligned with the pre-integration site, absent the *Alu*. Therefore, we performed Sanger DNA sequencing on the *Alu* present PCR amplicon from *S. apella* (UAM-46596) and aligned these results to confirm the presence of a shared insertion (Supplementary File S3). The finding of a relatively young *Alu* insertion from genus *Cebus* that is polymorphic within genus *Sapajus* is unexpected but not impossible given that the *Cebus* dataset was not filtered against [GSC_monkey_1.0] and the integration of this locus occurred prior to the *Cebus*/*Sapajus* divergence.

If these experimental results are extrapolated, 30 insertion polymorphisms identified among 132 analyzed by PCR correspond to a *Cebus* species level insertion polymorphism rate of 22.7%, suggesting that there are potentially over 350 insertion polymorphisms available within this dataset of 1607 young *Alu* elements. However, our relatively small DNA sample size containing only a few species limits the utility of further PCR for this study. Alternatively, we provide oligonucleotide PCR primer sequences for an additional 632 young candidate loci employed by other research groups with access to a larger number of species and individuals. These oligonucleotides for PCR and their predicted amplicon sizes for *Cebus* and closely related outgroups are available in Supplementary File S1.

The *S. apella* [GSC_monkey_1.0] genome assembly (first available in 2019) was analyzed for full-length *Alu* elements, and the output was then filtered against the [Cebus_imitator-1.0] genome using BLAT [49], resulting in 29,554 putatively *Sapajus* lineage-specific *Alu* elements. This output was subsequently filtered using a custom Python script (available on link https://github.com/t-beck; accessed on 4 March 2022) to BLAT the genomes of human, *C. imitator*, marmoset, owl monkey, and squirrel monkey in a single program, resulting in 8135 *Alu* elements lineage-specific to the *S. apella* genome. We identified 1170 of these as young (≤2% sequence divergence from their consensus). The local RepeatMasker output was further screened to ensure the position of the *Alu* was near the center (i.e., 600 bp) of the target sequence to represent the intended insertion, producing our reported dataset of 678 young *Alu* insertions, representing 20 different *Alu* subfamilies, for PCR. Genomic coordinates for these loci are shown in Supplementary File S2. We obtained oligonucleotides for PCR for 214 of these candidate loci. Following assessment by NCBI Primer Blast [52], we report oligonucleotide primer pairs along with their predicted amplicon sizes for 110 *Sapajus Alu* insertions (Supplementary File S2).

**Figure 3:**
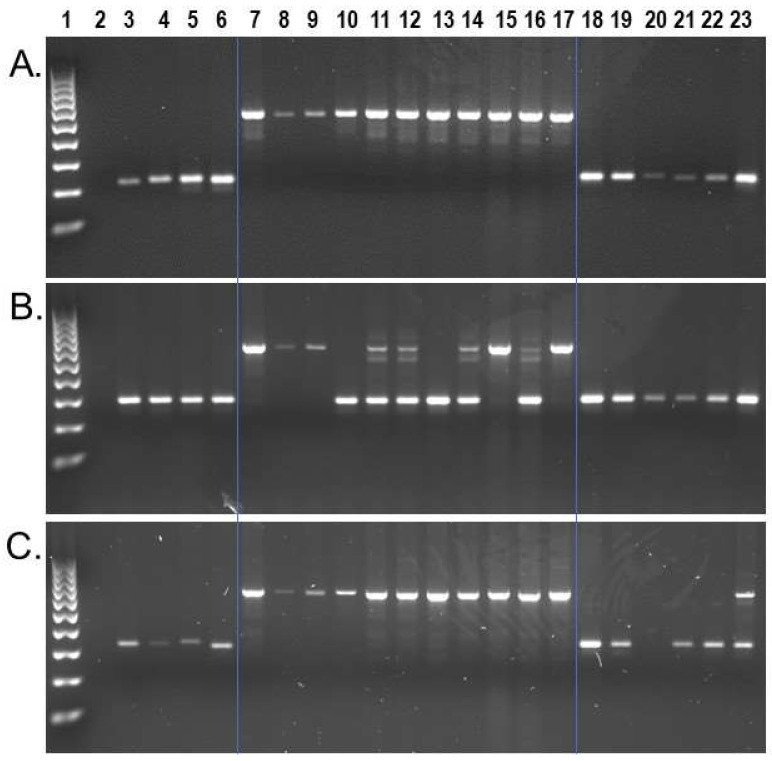
*S. apella* genomic *Alu* insertion polymorphisms. Lanes: 1 100 bp DNA ladder, 2 TLE (negative control), 3 Human (HeLa), 4 *C. jacchus* (Common marmoset), 5 *S. s. sciureus* (Common squirrel monkey), 6 *A. trivirgatus* (Three striped owl monkey), 7–17 *S. apella,* 18 *C. imitator*, 19–20 *C. capucinus*, 21–22 *C. albifrons*, 23 *C. a. albifrons*. (**A**) Locus # 984.99, the *Alu* element is present in all *Sapajus* individuals (~528 bp) and absent in all *Cebus* samples (~227 bp fragment); (**B**) Locus #978.25, the *Alu* element is polymorphic among *Sapajus* individuals (~604 bp and ~292 bp DNA fragments) and absent in *Cebus* samples; (**C**) Locus #944.49, the *Alu* element is present in all *Sapajus* individuals (~671 bp fragment), and heterozygous present (~671 bp and ~341 bp DNA fragments) in individual KB-4207, *C. a. albifrons*. Blue lines superimposed on gel images visually separate *Cebus*, *Sapajus* and outgroups.

Wet bench locus-specific PCR analyses for a subset of n = 74 identified n = 50 as homozygous present in all *Sapajus* samples, while homozygous absent in all *Cebus* samples on our DNA sample panel (Figure 3a). An additional n = 19 displayed polymorphic patterns for insertion presence/absence among the eleven *S. apella* samples while remaining homozygous absent in all *Cebus* samples (Figure 3b). Interestingly, we also identified two *Alu* insertions that, while being homozygous present in all eleven *S. apella* samples, were also confirmed to be shared by *C. albifrons* sample KB-4207. Locus #944.49 (Figure 3c) was confirmed to be the same as the target *Alu* by Sanger chain-termination DNA sequencing, and locus #961.24 was confirmed to have the same *Alu* insertion present in the *C. albifrons* genome assembly, [CebAlb_V1_BIUU] (Supplementary File S3). Both insertions are absent in [Cebus_imitator-1.0] and other *Cebus* species analyzed. A third locus, #993.63, displayed a similar gel electrophoresis pattern to locus #944.49; however, in this case, Sanger DNA sequencing revealed that the *Alu* present PCR amplicon in sample KB-4207, *C. albifrons*, represented a different *Alu* element ~70 bp away from the target insertion. We identified a second incidence of a ‘near-parallel insertion’ in locus #954.16, in which PCR results implied that the *Sapajus* target *Alu* insertion was also homozygous present in two *Cebus* samples, Cc_AM_T3 used for the *C. imitator* reference genome and *C. capucinus* sample UF-31995. In this case, Sanger DNA sequencing revealed that the two *Cebus* samples contained a different *Alu* element ~100 bp away and in the opposite orientation from the target insertion (Supplementary File S3). The homologous region of the *C. imitator* genome for this locus is located on an unplaced genomic scaffold and displays the *Alu* absent pre-integration sequence. Sequence alignments are shown in Supplementary File S3. Only one locus of the n = 74 analyzed by PCR, locus #942.22, also located on an unplaced genomic scaffold in [Cebus_imitator-1.0], appears shared by all capuchins on our DNA sample panel, indicating that our strategy to select *Sapajus* specific insertions was generally effective.

The combined experimental PCR data for these capuchin *Alu* datasets provides 104 insertion polymorphisms informative at the genus level as either *Cebus*-indicative (n = 54) or *Sapajus*-indicative (n = 50) genetic markers. Because of their high allele frequency for the respective genus, these genus indicative *Alu* elements could assist in clarifying the identity of archival specimens that may have been labeled simply as ‘Cebus monkey’ prior to the use of more refined taxonomic designations. They also provide strong support for *Cebus* and *Sapajus* being genetically separate genera, given that ~40% of the capuchin lineage-specific *Alu* insertions ascertained from the *C. imitator* genome were completely absent from *Sapajus* samples. PCR validation experiments provide an additional 51 *Alu* insertion polymorphisms potentially informative for capuchin phylogeny, with intermediate allele frequency among *Cebus* species (n = 29 loci), among *Sapajus* individuals (n = 19 loci), or perhaps both (n = 3). *Cebus*_locus #9 and *Sapajus* loci #944.49 and #961.24 integrated into the capuchin genome prior to the divergence of the two genera but remained variable for insertion presence/absence across taxa. The polymorphism rate within the *Alu* datasets reported here is ~25%, providing potentially hundreds of additional insertion polymorphisms to study capuchin phylogeny and conservation genetics.

## 4. Discussion

This study provides an extensive dataset of recently integrated *Alu* mobile elements in the capuchin lineage and demonstrates their phylogenetically diagnostic utility. Even with the large number of *Alu* insertion polymorphism reported here, these data are still subject to some ascertainment bias towards the two genome assemblies analyzed. The *Alu* elements ascertained from the [Cebus_imitator-1.0] genome were not computationally filtered against the genome assembly for *S. apella*, [GSC_monkey_1.0] and therefore were expected to be more broadly represented among capuchins. However, roughly 40% of those analyzed by PCR were absent in all *S. apella* samples. This provides strong support for the division of capuchins into the two genera as proposed by Lynch Alfaro et al. [1] and bolstered by Martins et al. [46]. It also implies some degree of reproductive restriction during their history, leading to genus level monophyly despite large areas of sympatry [6].

The diversification of capuchins into two separate lineages is believed to have started with *Sapajus* capuchins first, about 2.7 mya, and then slightly later for *Cebus* at around 2.1 mya [1], while the radiation of extant capuchin species has occurred relatively recently in the last 1–2 mya [2]. Studies of capuchin phylogenetic relationships using mitochondrial DNA [2,3] generally support these divergence estimates but find only limited support for any single topology of phylogenetic relationships within each genus. A study of *Sapajus* phylogeny [5] using ultraconserved elements (UCEs) [57] showed strong support for *S. xanthosternos*, *S. nigritus*, and *S. robustus* as defined branches within the clade, but all other species of robust capuchins grouped together. These presently unresolved polytomies within capuchin phylogeny are largely attributed to an extremely rapid speciation and dispersal process that occurred 2–3 mya [2,6]. Such events can lead to incomplete lineage sorting (ILS) of phylogenomic markers that remained unfixed in the population during speciation and later become randomly fixed or extinct in emerging species [58,59].

Contrasting evidence to genus level monophyly and a surprising finding of this study was the discovery of two independent *Alu* insertions shared by members of *S. apella* and *C. albifrons*, to the exclusion of [Cebus_imitator-1.0] and *C. capucinus*. One of these was confirmed to be shared in the *C. albifrons* genome assembly [CebAlb_V1_BIUU], eliminating the possibility that this result was due to sample mix-up. The other was validated by DNA sequencing. These data suggest that *S. apella* and *C. albifrons* are phylogenetically more closely related to each other than *S. apella* is to the other *Cebus* species. This conclusion is unlikely to be true, given the morphological differences and that no studies to date provide substantiating evidence in support of this relationship. This situation could result from ILS as described above or possibly due to introgression. A possible hybrid zone has been reported for *S. robustus* and *S. nigritus* [60], and others are suspected to exist in many contact zones between multiple species [2]. Known populations of *C. albifrons* and *S. apella* are sympatric in the North Amazon [2], contributing to this possibility. Data for these two *Alu* insertion polymorphisms from a much larger sample size comprised of representatives of all capuchin species are required to resolve this issue.

This example of a confounding topology highlights the primary limitation of this study. The DNA sample panels used in this study were relatively small, both in the number of individuals and the number of species available. They did not include any samples for two *Cebus* species, *C. kaapori* and *C. olivaceus*. In addition, the only robust capuchin species represented was *S.* *apella,* and DNA sample panels lacked the other seven recognized *Sapajus* species. However, given that all *Sapajus* samples were acquired having the label ‘*Cebus apella*’ and the name *Cebus apella* was universally used for all robust capuchins until recently, it is possible that some samples could be from other *Sapajus* species that lacked independent designations at the time of collection. This scenario would create an over-estimate of the genetic variance within *S. apella* from these data. Furthermore, only one DNA sample (Cc_AM_T3) was originally from a wild population. All the other samples were derived from captive subjects, which could result in an under-estimate of the actual variation among individuals and populations in their natural environment. Another potential confounding factor of captive capuchins is the possibility of having cross-species hybrids due to their breeding history while in captivity [61].

Another interesting finding of this study was the identification of two confirmed cases of a ‘near parallel insertion’ among capuchin genomes. This situation occurs when an independent retrotransposon inserts within the sequence span of the PCR amplicon, possibly confounding interpretation of the results. Near parallel insertions have previously been reported to be rare in human genomes [59] and easily resolved by DNA sequencing as they were here. However, the number of capuchin lineage-specific *Alu* insertions (~9000) is considerably higher than the number of reported human-specific *Alu* elements (~5000) [62] over roughly the same 6 my evolutionary time frame. A higher *Alu* mobilization rate increases the likelihood of near-parallel insertions occurring. Future researchers utilizing the datasets reported in this study will need to be cognizant when interpreting PCR results.

The future availability of more genome assemblies for additional capuchin species will help alleviate many confounding issues and make it easier to check for near parallel insertions. The set of young *Alu* elements ascertained from the *S. apella* genome [GSC_monkey_1.0] was filtered against the *C. imitator* genome and may be more specific for targeting allele variance among *Sapajus* species. Still, one individual from a single species within each genus may not represent the entire genus. Having an adequate number of DNA samples for all species would help to refine capuchin phylogenetic relationships and expose if multiple alternative topologies remain likely. Unresolved polytomies, while using unidirectional *Alu* insertions as phylogenetic markers, would be indicative of ancient and/or ongoing gene flow and reticulation among *Sapajus* species or perhaps more broadly with *Cebus* as well.

## 5. Conclusions

This study provides the most comprehensive dataset of phylogenetically diagnostic *Alu* insertion polymorphisms for the capuchin lineage reported to date. It shows that multiple *Alu* subfamilies have evidence of recent mobilization within capuchin genomes. These datasets of autosomal-based *Alu* elements that have a unidirectional mode of evolution will provide researchers with a much-needed additional resource for the study of capuchin phylogenetic relationships and for conservation strategies.

## Data Availability

The algorithms used in this study are available on GitHub (https://github.com/t-beck; accessed on 4 March 2022). The Supplementary data files are available on the online version of this paper and through the Batzer Lab website under publications, https://biosci-batzerlab.biology.lsu.edu/; accessed on 4 March 2022.

## Supplementary Materials

The following supporting information can be downloaded at: https://www.mdpi.com/article/10.3390/genes13040572/s1. Supplementary File S1 is an Excel file containing the RepeatMasker output for the *Alu* elements ascertained from [Cebus_imitator-1.0] and separate worksheets for PCR primers, DNA samples and genotypes; Supplementary File S2 is an Excel file containing the RepeatMasker output for the *Alu* elements ascertained from [GSC_monkey_1.0] and separate worksheets for PCR primers, DNA samples and genotypes; Supplementary File S3 is a *.gb file constructed in BioEdit (Hall 1999) showing DNA sequence alignments for five *Alu* insertion candidates with ambiguous PCR results; also available as a *.pdf PDF document file.
