# Supplementary material for: Recently Integrated Alu Elements in Capuchin Monkeys: A Resource for Cebus/Sapajus Genomics
Source: Genes (Basel). 2022 Mar 24;13(4):572. doi: 10.3390/genes13040572 (PMC9030454; doi:10.3390/genes13040572)
Supplement: Supplementary file 1 [file genes-13-00572-s001.zip › Batzer_Supplementary_File3_alignments_doc.pdf]

```
                                                  10        20        30        40        50        60        70        80        90       100
                                          ....|....|....|....|....|....|....|....|....|....|....|....|....|....|....|....|....|....|....|....|
Cebus-9-Saimiri genome                    GCAGTAAAATGCTTCAGCAAACagtaaaattttgttttacttttagttttatttttctgagaagttagattttgaaggcatccatcacttaataaatgaa
Cebus-9-Cebus imitator genome             GCAGTAAAATGCTTCAGCAAACAGTAAAATTTTGTTTTACTTTTAGTTTTATTTTCCAGAGAAGTTAGATTTTGAGAGCACCCATCACTTAATAAACCAA
Cebus-9-Sapajus apella genome             GCAGTAAAATGCTTCAGCAAACAGTAAAATTTTGTTTTACTTTTAGTTTTATTTTCCAGAGAAGATAGATTTTGAGGGCACCCATCACTAAATAAACCAA
Cebus-9-UAM46596-F                        ~~~~~~~~~~~~~~~~~~~~~~~~~~~~~~~~~~~~~~~~~~~~~~~~~~~~~~~~~~~~~~~~~~~~~~~~~~~~~~~~~~~ATCACTTAATAAACCAA
Cebus-9-UAM46596-SIntR1-rc                ~~~~~~~~~~~~TTCAGCAAACAGTAAAATTTTGTTTTACTTTTAGTTTTATTTTCCAGAGAAGATAGATTTTGAGGGCACCCATCACTTAATAAACCAA
Cebus-9-UAM46596-IntF1                    ~~~~~~~~~~~~~~~~~~~~~~~~~~~~~~~~~~~~~~~~~~~~~~~~~~~~~~~~~~~~~~~~~~~~~~~~~~~~~~~~~~~~~~~~~~~~~~~~~~~~
Cebus-9-UAM46596-R-rc                     ~~~~~~~~~~~~~~~~~~~~~~~~~~~~~~~~~~~~~~~~~~~~~~~~~~~~~~~~~~~~~~~~~~~~~~~~~~~~~~~~~~~~~~~~~~~~~~~~~~~

                                                 110       120       130       140       150       160       170       180       190       200
                                          ....|....|....|....|....|....|....|....|....|....|....|....|....|....|....|....|....|....|....|....|
Cebus-9-Saimiri genome                    atcgagagaaagaaatcactgaaaaagaaaa~tgtaaactttgattgactctctgcctttgtgtagaaagcatgactgat~~~~~~~~~~~~~~~~~~~~~
Cebus-9-Cebus imitator genome             ATCAAGAGAAAGAAGGCACTGAAAAAAAAAA~TGTAAACTTTGATTGACTCTCCGCTTTTGTGTAGAAAACATGATTGATGGGCCGGGCGCGGTGGCTCA
Cebus-9-Sapajus apella genome             ATCAAGAGAAAGAAGACACTGAAAAAAAAAAATGTAAACTTTGATTGACTCTCCGCTTTTGTGTAGAAAACATGATTGAT~~~~~~~~~~~~~~~~~~~~~
Cebus-9-UAM46596-F                        ATCAAGAGAAAGAAGACACTGAAAAAAAAAA~TGTAAACTTTGATTAACTCTCCGCTTTTGTGTAGAAAACATGATTGATGGGCCGGGCGCGGTGGCTCA
Cebus-9-UAM46596-SIntR1-rc                ATCAAGAGAAAGAAGACACTGAAAAAAAAAA~TGTAAACTTTGATTAACTCTCCGCTTTTGTGTAGAAAACATGATTGATGGGCCGGGCGCGGTGGCTCA
Cebus-9-UAM46596-IntF1                    ~~~~~~~~~~~~~~~~~~~~~~~~~~~~~~~~~~~~~~~~~~~~~~~~~~~~~~~~~~~~~~~~~~~~~~~~~~~~~~~~~~~~~~~~~~~~~~~~~~~~
Cebus-9-UAM46596-R-rc                     ~~~~~~~~~~~~~~~~~~~~~~~~~~~~~~~~~~~~~~~~~~~~~~~~~~~~~~~~~~~~~~~~~~~~~~~~~~~~~~~~~~~~~~~~~~~~~~~~~~~~

                                                 210       220       230       240       250       260       270       280       290       300
                                          ....|....|....|....|....|....|....|....|....|....|....|....|....|....|....|....|....|....|....|....|
Cebus-9-Saimiri genome                    ~~~~~~~~~~~~~~~~~~~~~~~~~~~~~~~~~~~~~~~~~~~~~~~~~~~~~~~~~~~~~~~~~~~~~~~~~~~~~~~~~~~~~~~~~~~~~~~~~~~~
Cebus-9-Cebus imitator genome             CGCCTGTAATCCCAGCACTTTGGGAGGCCGAGGCGGGTGGATCACGAAGTCAAGAGATCGAGACCATCCTGGTCAACATGGTGAAACCCCGTCTCTACTA
Cebus-9-Sapajus apella genome             ~~~~~~~~~~~~~~~~~~~~~~~~~~~~~~~~~~~~~~~~~~~~~~~~~~~~~~~~~~~~~~~~~~~~~~~~~~~~~~~~~~~~~~~~~~~~~~~~~~~~
Cebus-9-UAM46596-F                        CGCTTGTAATCCCAGCACTTTGGGAGGCCGAGGCGGGTGGATCACAAGGTCAAGAGCTCGAGACCATCCTGGTCAACATGGTGAAACCCCGTCTCTACTA
Cebus-9-UAM46596-SIntR1-rc                CGCTTGTAATCCCAGCACTTTGGGAGGCCGAGGCGGGTGGATCACAAGGTCAAGAGCTCGAGACCATCCTGGTCAACATGGTGAAACCCCGTCTCTACTA
Cebus-9-UAM46596-IntF1                    ~~~~~~~~~~~~~~~~~~~~~~~~~~~~~~~~~~~~~~~~~~~~~~~~~~CAAGAGCTCGAGACCATCCTGGTCAACATGGTGAAACCCCGTCTCTACTA
Cebus-9-UAM46596-R-rc                     ~~~~~~~~~~~~~~~~~~~~~~~~~~~~~~~~~~~~~~~~~~~~~~~~~~~~~~~~~~~~~~~~~~~~~~~~~~~~~~~~~~~~~~~~~~~~~~~~~~~~

                                                 310       320       330       340       350       360       370       380       390       400
                                          ....|....|....|....|....|....|....|....|....|....|....|....|....|....|....|....|....|....|....|....|
Cebus-9-Saimiri genome                    ~~~~~~~~~~~~~~~~~~~~~~~~~~~~~~~~~~~~~~~~~~~~~~~~~~~~~~~~~~~~~~~~~~~~~~~~~~~~~~~~~~~~~~~~~~~~~~~~~~~~
Cebus-9-Cebus imitator genome             AAAATACAAAAAATTAGCTGGGCATGGTGGTGCGTGCCTGTAATCCCAGCTGCTCAGGAGGCTGAGGCAGGAGAATTGCCTGAACCCAGGAGGCGGAGGT
Cebus-9-Sapajus apella genome             ~~~~~~~~~~~~~~~~~~~~~~~~~~~~~~~~~~~~~~~~~~~~~~~~~~~~~~~~~~~~~~~~~~~~~~~~~~~~~~~~~~~~~~~~~~~~~~~~~~~~
Cebus-9-UAM46596-F                        AAAATACAAAAAATTAGCTGGGCATGGTGGTGCGTGCCTGTAATCCCAGCTACTTAGGAGGCTGAGGCAGGAGAATTGCTTGAACCCAGGAGGCGGAGGT
Cebus-9-UAM46596-SIntR1-rc                AAAATACAAAAAATTAGCTGGGCATGGTGGTGCGTGCCTGTAATCCCAGC
Cebus-9-UAM46596-IntF1                    AAAATACAAAAAATTAGCTGGGCATGGTGGTGCGTGCCTGTAATCCCAGCTACTTAGGAGGCTGAGGCAGGAGAATTGCTTGAACCCAGGAGGCGGAGGT
Cebus-9-UAM46596-R-rc                     ~~~~~~~~~~~~~~~~~~~~~~~~~~~~~~~~~~~~~~~~~~~~~~~~~~~~~~~~~~~~~~~~~~~~~~~~~~~~~~~~~~~~~~~~~~~~~~~~~~~~

                                                 410       420       430       440       450       460       470       480       490       500
                                          ....|....|....|....|....|....|....|....|....|....|....|....|....|....|....|....|....|....|....|....|
Cebus-9-Saimiri genome                    ~~~~~~~~~~~~~~~~~~~~~~~~~~~~~~~~~~~~~~~~~~~~~~~~~~~~~~~~~~~~~~~~~~~~~~~~~~~~~~~~~~~~~~~~~~~~tttcttca
Cebus-9-Cebus imitator genome             TGCGGTGAGCCGAGATCGTGCCATTGCACTCCAGCCTGGGTAACAAGAGCAAAACTCCGTCTCAAAAAAAAAAAAAAAGAAAACATGATTGATTTTCTTCA
Cebus-9-Sapajus apella genome             ~~~~~~~~~~~~~~~~~~~~~~~~~~~~~~~~~~~~~~~~~~~~~~~~~~~~~~~~~~~~~~~~~~~~~~~~~~~~~~~~~~~~~~~~~~~~TTTCTTCA
Cebus-9-UAM46596-F                        TGCGGTGAGCCGAGATCATGCCATTGCACTCCAGCCGGGGTAACAAGAGCAAAACTCCGTCTCAAAAAAAAAAAAAAAAAAAAAAAAA
Cebus-9-UAM46596-SIntR1-rc
Cebus-9-UAM46596-IntF1                    TGCGGTGAGCCGAGATCATGCCATTGCACTCCAGCCGGGGTAACAAGAGCAAAACTCCGTCTCAAAAAAAAAAAAAAAAAAAAAAAAA
Cebus-9-UAM46596-R-rc                     ~~~~~~~~~~~~~~~~~~~~~~~~~~~~~~~~~~~~~~~~~~~~~~~~~~~~~~~~~~~~AAAAAAAAAAAAAAAAAAAAAGAAAACATGATTGATTTTCTTCA

                                                 510       520       530       540       550       560       570       580       590       600
                                          ....|....|....|....|....|....|....|....|....|....|....|....|....|....|....|....|....|....|....|....|
Cebus-9-Saimiri genome                    ggctatttttttcccatttttccgagtgtgtagtccattactcatcaaatttggtttcagaaagattctgaaaattcctttaatcttttaaaagctaaaga
Cebus-9-Cebus imitator genome             GGTTATTTTTTTCC~ATTTTTCCGAGTGTGTAGTCCATTACTCATCAAATTTGGTTTCAGAAAGAATCTGAAAATTCCTTTAATCTTTTAAAAGCTAAAGA
Cebus-9-Sapajus apella genome             CGTTATTTTTTTCC~ATTTTTCCGAGTGTGTAGTCCATTACTCATCAAATTTGGTCTCAGAAAGATTCTGAAAATTCCTTTAATCTTTTAAAAGCTAAAGA
Cebus-9-UAM46596-F
Cebus-9-UAM46596-SIntR1-rc
Cebus-9-UAM46596-IntF1
Cebus-9-UAM46596-R-rc                     GGTTATTTTTTTCC~ATTTTTCCGAGTGTGTAGTCCATTACTCATCAAATTTGGTTTCA
```

```
                                       210       220       230       240       250       260       270       280       290       300
                                ....|....|....|....|....|....|....|....|....|....|....|....|....|....|....|....|....|....|....|....|
Sapajus-944.49-S. apella genome TTATGTACAACTTGGAAGTAATAATATTTATAAAGTTGCTGGATTTAAATGTGACAAACTAGGAAATGAAATTAGTACATTATACAGTATTTAGTTTGTA
Sapajus-944.49-C. imitator geno TTATGTACAAATTGGAAGTAATAATATTTATAAAGTTGCTGGATTTAAATGAGACAAACTAGAAAATGAAATTAGTACATTATACAGTATTTAGTTTGTA
Sapajus-944.49-CebAlb_sc 319371 ~~~~~~~~~~~~~~~~~~~~~~~~~~~~~~~~~~~~~~~~~~~~~~~~~~~~~~~~~~~~~~~~~~~~~~~~~~~~~~~~~~~~~~~~~~~~~~~~~~~~
Sapajus-944.49-KB4207-F         ~~~~~~~~~~~~~~~~~~~~~~~~~~~~~~~~~~~~~~~~~~~~~~~~~~~~~~~~~~~~~~~~~~~~~~~~~~~~~~~~~~~~~~~~~~~~~~~~~~~~
Sapajus-944.49-KB4207-IntF1     ~~~~~~~~~~~~~~~~~~~~~~~~~~~~~~~~~~~~~~~~~~~~~~~~~~~~~~~~~~~~~~~~~~~~~~~~~~~~~~~~~~~~~~~~~~~~~~~~~~~~
Sapajus-944.49-KB4207-SIntR1rc  ~~~~~~~~~~~~~~~~~~~~~~~~~~~~~~~~~~~~~~~~~~~~~~~~~~~~~~~~~~~~~~~~~~~~~~~~~~~~~~~~~~~~~~~~~~~~~~~~~~~~
Sapajus-944.49-KB4207-R-rc      ~~~~~~~~~~~~~~~~~~~~~~~~~~~~~~~~~~~~~~~~~~~~~~~~~~~~~~~~~~~~~~~~~~~~~~~~~~~~~~~~~~~~~~~~~~~~~~~~~~~~
                                       310       320       330       340       350       360       370       380       390       400
                                ....|....|....|....|....|....|....|....|....|....|....|....|....|....|....|....|....|....|....|....|
Sapajus-944.49-S. apella genome GACCTTATTTTTATTATTATTTAAATATTGATATGTATATTTTGGACATTAGAGCTTGGTTTTGTCTATCTCAAACCAGAAGTTTTAAACTACCCTGCAG
Sapajus-944.49-C. imitator geno GCCCTTATTTTTATTATTATTTAAATATTGATATGTATATTTTGGACATTAGAGCTTGGTTTTGTCTATCTCAAACCAGAAGTTTTAAACTACCCTGCAG
Sapajus-944.49-CebAlb_sc 319371 ~~~~~~~~~~~~~~~~TTATTTAAATATTGATATGTATATTTTGGACATTAGAGCTTGGTTTTGTCTATCTCAAACCAGAAGTTTTAAACTACCCTGCAG
Sapajus-944.49-KB4207-F         ~~~~~~~~~~~~~~~~~~~~~~~~~~~~~~~~~~~~~~~~~~~~~~~~~~~~~~~~~~~~~~~~~~~~~~~~~~~~~~~~~~~~~~~~~~~~~~~~~~~~
Sapajus-944.49-KB4207-IntF1     ~~~~~~~~~~~~~~~~~~~~~~~~~~~~~~~~~~~~~~~~~~~~~~~~~~~~~~~~~~~~~~~~~~~~~~~~~~~~~~~~~~~~~~~~~~~~~~~~~~~~
Sapajus-944.49-KB4207-SIntR1rc  ~~~~~~~~~~~~~~~~~~~~~~~~~~~~~~~~~~~~~~~~~~~~~~~~~~~~~~~~~~~~~~~~~~~~~~~~~~~~~~~~~~~~~~~~~~~~~~~~~~~~
Sapajus-944.49-KB4207-R-rc      ~~~~~~~~~~~~~~~~~~~~~~~~~~~~~~~~~~~~~~~~~~~~~~~~~~~~~~~~~~~~~~~~~~~~~~~~~~~~~~~~~~~~~~~~~~~~~~~~~~~~
                                       410       420       430       440       450       460       470       480       490       500
                                ....|....|....|....|....|....|....|....|....|....|....|....|....|....|....|....|....|....|....|....|
Sapajus-944.49-S. apella genome AAACTTGATGGGAGAAGAGCTGGAATGCCTCCCTCCAGCTGTACCTCATGTTTCAGAATAGAATTCATCCTGTTGTAATGTGTCAGCTTAGGCAGGTCAG
Sapajus-944.49-C. imitator geno AAACTTGATGGGAGAAGAA~TGGAATGCCTCCCTCCAGCTGTACGTCATGTTTCAGAATAGAATTCATCCTGTTGTAATGTGTCAGCTTAGGCAGGCCAG
Sapajus-944.49-CebAlb_sc 319371 AAACTTGATGGGAGAAGAA~TGGAATGCCTCCCTCCAGCTGTACGTCATGTTTCAGAATAGAATTCATCCTGTTGTAATGTGTCAGCTTAGGCAGGCCAG
Sapajus-944.49-KB4207-F         ~~~~~~~~~~~~~~~~~~~~~~~~~~~~~~~~~~~~~~~~~~~~~~~~~~~~~~~~~~~~~~~~~~~~~~~~~~~~~~~GTGTCAGCTTAGGCAGGTCAG
Sapajus-944.49-KB4207-IntF1     ~~~~~~~~~~~~~~~~~~~~~~~~~~~~~~~~~~~~~~~~~~~~~~~~~~~~~~~~~~~~~~~~~~~~~~~~~~~~~~~~~~~~~~~~~~~~~~~~~~~~
Sapajus-944.49-KB4207-SIntR1rc  ~~~~~~~~~~~~~~~~~~~~~~~~~~~~~~~~~~~~~~~~~~~~~~~~~~~~~~~~~~~~~~~~~~~~~~~~~~~~~~~~~~~~~~~~~~~~~~~~~~~~
Sapajus-944.49-KB4207-R-rc      ~~~~~~~~~~~~~~~~~~~~~~~~~~~~~~~~~~~~~~~~~~~~~~~~~~~~~~~~~~~~~~~~~~~~~~~~~~~~~~~~~~~~~~~~~~~~~~~~~~~~
                                       510       520       530       540       550       560       570       580       590       600
                                ....|....|....|....|....|....|....|....|....|....|....|....|....|....|....|....|....|....|....|....|
Sapajus-944.49-S. apella genome CTTCTTGAAAGGCAACGCCATAGTTTGGGCTTAGATACAAGTCAATTATATCAATTCCAAGAAAAAAAAAAAAGAATATCTTTAAAAATCAATCTCAAGG
Sapajus-944.49-C. imitator geno CTTCTTGAAAGGCAATGCCATAGTTTGGGCTTAGATACAAGTCAATTATATCAATTCCAAGAAAAAAAAAA~~GAATAGCTTTAAAAATCAATCTCAA~~
Sapajus-944.49-CebAlb_sc 319371 CTTCTTGAAAGGCAATGCCATAGTTTGGGCTTAGATACAAGTCAATTATATCAATTCCAAGAAAAAAAAAA~~GAATAGCTTTAAAAATCAATCTCAA~~
Sapajus-944.49-KB4207-F         CTTCTTGAAAGGCAATGCCATAGTTTGGGCTTAGATACAAGTCAATTATATCAATTCCAAGAAAAAAAAAA~AAAATATCTTTAAAAATCAATCTCAAGG
Sapajus-944.49-KB4207-IntF1     ~~~~~~~~~~~~~~~~~~~~~~~~~~~~~~~~~~~~~~~~~~~~~~~~~~~~~~~~~~~~~~~~~~~~~~~~~~~~~~~~~~~~~~~~~~~~~~~~~~~~
Sapajus-944.49-KB4207-SIntR1rc  ~~~~~~~~~~~~~~~~~~~~~~~~~~~~~~~~~~~~~~~~~~~~~~~~~~~~~~~~~~~~~~AAAAAAAAAAAGAATATCTTTAAAAATCAATCTCAAGG
Sapajus-944.49-KB4207-R-rc      ~~~~~~~~~~~~~~~~~~~~~~~~~~~~~~~~~~~~~~~~~~~~~~~~~~~~~~~~~~~~~~~~~~~~~~~~~~~~~~~~~~~~~~~~~~~~~~~~~~~~

                                       610       620       630       640       650       660       670       680       690       700
                                ....|....|....|....|....|....|....|....|....|....|....|....|....|....|....|....|....|....|....|....|
Sapajus-944.49-S. apella genome GGCCGGGCGCGGTGGCTCACGCCTGTAATCCCAGCACTTTGGGAGGCCGAGGCGGGTGGATCATGAGGTCAAGAGATCGAGACCATCCTGGTCAACATGG
Sapajus-944.49-C. imitator geno ~~~~~~~~~~~~~~~~~~~~~~~~~~~~~~~~~~~~~~~~~~~~~~~~~~~~~~~~~~~~~~~~~~~~~~~~~~~~~~~~~~~~~~~~~~~~~~~~~~~~
Sapajus-944.49-CebAlb_sc 319371 ~~~~~~~~~~~~~~~~~~~~~~~~~~~~~~~~~~~~~~~~~~~~~~~~~~~~~~~~~~~~~~~~~~~~~~~~~~~~~~~~~~~~~~~~~~~~~~~~~~~~
Sapajus-944.49-KB4207-F         GGCCAGGCGCGGTGGCTCACGCCTGTAATCCCAGCACTTTGGGAGGCCAAGGCGGGTGGATCATGAGGTCAAGGGATTGAAACCATCCTGGTCAACATGG
Sapajus-944.49-KB4207-IntF1     ~~~~~~~~~~~~~~~~~~~~~~~~~~~~~~~~~~~~~~~~~~~~~~~~~~~~~~~~~~~~~~~~GAGGTCA~GGGATTGAGACCATCCTGGTCAACATGG
Sapajus-944.49-KB4207-SIntR1rc  GGCCAGGCGCGGTGGCTCACGCCTGTAATCCCAGCACTTTGGGAGGCCGAGGCGGGTGGATCATGAGGTCAAGGGATTGAGACCATCCTGGTCAACATGG
Sapajus-944.49-KB4207-R-rc      ~~~~~~~~~~~~~~~~~~~~~~~~~~~~~~~~~~~~~~~~~~~~~~~~~~~~~~~~~~~~~~~~~~~~~~~~~~~~~~~~~~~~~~~~~~~~~~~~~~~~
                                       710       720       730       740       750       760       770       780       790       800
                                ....|....|....|....|....|....|....|....|....|....|....|....|....|....|....|....|....|....|....|....|
Sapajus-944.49-S. apella genome TGAAACCCCGTCTCTACTAAAAATACAAAAAATTAGCTGGGCATGGTGGCACATGCCTGTAATCCCAGCTACTCAGGAGGCTGAGACAGGAGAATTGCCT
Sapajus-944.49-C. imitator geno ~~~~~~~~~~~~~~~~~~~~~~~~~~~~~~~~~~~~~~~~~~~~~~~~~~~~~~~~~~~~~~~~~~~~~~~~~~~~~~~~~~~~~~~~~~~~~~~~~~~~
Sapajus-944.49-CebAlb_sc 319371 ~~~~~~~~~~~~~~~~~~~~~~~~~~~~~~~~~~~~~~~~~~~~~~~~~~~~~~~~~~~~~~~~~~~~~~~~~~~~~~~~~~~~~~~~~~~~~~~~~~~~
Sapajus-944.49-KB4207-F         TGAAACCCCGTCTCTACTAAAAATACAAAAAATCACCTGGG
Sapajus-944.49-KB4207-IntF1     TGAAACCCCGTCTCTACTAAAAATACAAAAAATCAGCTGGGCATGGTGGCGCGTGCCTGTAGTCCCAGCTACTCAGGAGGCTGAGGCAGGAGAATTACCT
Sapajus-944.49-KB4207-SIntR1rc  TGAAACCCCGTCTCTACTAAAAATACAAAAAATCAGCTGGGCATGGTGGCGCGTGCCTGTAGTCCCAGCTACTCAGGAGG
Sapajus-944.49-KB4207-R-rc      ~~~~~~~~~~~~~~~~~~~~~~~~~~~~~~~~~~~~~~~~~~~~~~~~~~~~~~~~~~~~~~~~~~~~~~~~~~~~~~~~~~~~~~~~~~~~~~~~~~~~
```

```
                                           810       820       830       840       850       860       870       880       890       900
                                   ....|....|....|....|....|....|....|....|....|....|....|....|....|....|....|....|....|....|....|....|
Sapajus-944.49-S. apella genome    GAACCCGGGAGGCGGAGGTTGCGGTGAGCCGAGATCGCGCCATTGCACTCCAGCCTGGGCAACAAGAGCGAAACTCCGTCTCAAAAACAAAAACAAAAAC
Sapajus-944.49-C. imitator geno    ~~~~~~~~~~~~~~~~~~~~~~~~~~~~~~~~~~~~~~~~~~~~~~~~~~~~~~~~~~~~~~~~~~~~~~~~~~~~~~~~~~~~~~~~~~~~~~~~~~~~
Sapajus-944.49-CebAlb_sc 319371    ~~~~~~~~~~~~~~~~~~~~~~~~~~~~~~~~~~~~~~~~~~~~~~~~~~~~~~~~~~~~~~~~~~~~~~~~~~~~~~~~~~~~~~~~~~~~~~~~~~~~
Sapajus-944.49-KB4207-F
Sapajus-944.49-KB4207-IntF1        GAACCCAGGTGGTGGAGGTTGTGGTGAGCCGAGATCGCGCCATTGCACTCCAGCCTGGGTAACAAGAGCGAAACTCCGTCTCAAAAAAAAAAAAAATAAA
Sapajus-944.49-KB4207-SIntR1rc
Sapajus-944.49-KB4207-R-rc         ~~~~~~~~~~~~~~~~~~~~~~~~~~~~~~~~~~~~~~~~~~~~~~~~~~~~~~~~~~~~~~~~~~~~~~~~~~~~~~~~~~~~~~~~~~~~~~~~AAAA

                                           910       920       930       940       950       960       970       980       990      1000
                                   ....|....|....|....|....|....|....|....|....|....|....|....|....|....|....|....|....|....|....|....|
Sapajus-944.49-S. apella genome    AAAAACAACAAAAAAAATCTATCTCAAAATATCTCATTGAAGCTCTTGCTGCTTTGCTTCCTAGAAGAGGATTCTTTAGGTAGAGGGAGTTTCCTTCAGT
Sapajus-944.49-C._imitator geno    ~~~~~~~~~~~~~~~~~~~~~~~~~~~AATATCTCATTGAAGCTCTTGCTGCTTTGCTTCCTAGAAGAGGATTCTTTAGGTAGCAGGAGTTTCCTTCAGT
Sapajus-944.49-CebAlb_sc 319371    ~~~~~~~~~~~~~~~~~~~~~~~~~~~AATATCTCATTGAAGCTCTTGCTGCTTTGCTTCCTAGAAGAGGATTCTTTAGGTAGCAGGAGTTTCCTTCAGT
Sapajus-944.49-KB4207-F
Sapajus-944.49-KB4207-IntF1        AA
Sapajus-944.49-KB4207-SIntR1rc
Sapajus-944.49-KB4207-R-rc         AAAAAAAAAAATAATAATCAATCTCAAAATATCTCATTGAAGCTCTTGCTGCTTTGCTTCCTAGAAGAGGATTCTTTAGGTAGCGGGAGTTTCCTTCAGT
```

```
                                           410       420       430       440       450       460       470       480       490       500
                                   ....|....|....|....|....|....|....|....|....|....|....|....|....|....|....|....|....|....|....|....|
Sapajus-993.63-S. apella genome    TATTGTATCATGATCTACAGACTGAAGTTATTCTTCCACAGAAGCCTCTGTTGCCTGCGATGTGAGTTCTCTGAGGACAGTGACTCTggctcatttatta
Sapajus-933.63-C. albifrons_rc     ~~~~~~~~~~~~~~~~~~~~~~~~~~~~~~~~~~~~~~~~~~~~~~~~~~~~~~~~~~~~~~~~~~~~~TCTGAGGACAGTGACTCTGGCTCATTTATTA
Sapajus-993.63-KB4207-F            ~~~~~~~~~~~~~~~~~~~~~~~~~~~~~~~~~~~~~~~~~~~~~~~~~~~~~~~~~~~~~~~~~~~~~~~~~~~~~~~~~~~~~~~~~~~~~~~~~~~~
Sapajus-993.63-KB4207-SIntR1rc     ~~~~~~~~~~~~~~~~~~~~~~~~~~~~~~~~~~~~~~~~~~~~~~~~~~~~~~~~~~~~~~~~~~~~~~~~~~~~~~~~~~~~~~~~~~~~~~~~~~~~
Sapajus-993.63-KB4207-IntF1        ~~~~~~~~~~~~~~~~~~~~~~~~~~~~~~~~~~~~~~~~~~~~~~~~~~~~~~~~~~~~~~~~~~~~~~~~~~~~~~~~~~~~~~~~~~~~~~~~~~~~
Sapajus-993.63-KB4207-R-rc         ~~~~~~~~~~~~~~~~~~~~~~~~~~~~~~~~~~~~~~~~~~~~~~~~~~~~~~~~~~~~~~~~~~~~~~~~~~~~~~~~~~~~~~~~~~~~~~~~~~~~

                                           510       520       530       540       550       560       570       580       590       600
                                   ....|....|....|....|....|....|....|....|....|....|....|....|....|....|....|....|....|....|....|....|
Sapajus-993.63-S. apella genome    ttttcttaccaGCATGTAGGTAGACTGGAGTTCTTTAACAGCAGTGGCTTTTTGTTCTTAACTTGGCATCTATGTTGCTTAGAACATAGTAActatttaa
Sapajus-933.63-C. albifrons_rc     TTTTCTTACCAGCATGTAGGTAGACTGGAGTTCTTTAACAGCAGTGGCTTTTTGTTCTTAACTTGGCATCTATGTTGCTTAGAACATAGTAACTGTTTAA
Sapajus-993.63-KB4207-F            ~~~~~~~~~~~~~~~~~~~~~~~~~~~~~~~~~~~~~~~~~~~~~~~~~~~~~~~~~~~~~~TTGGCATCTATGTTGCTTAGAACATAGTAACTGTTTAA
Sapajus-993.63-KB4207-SIntR1rc     ~~~~~~TACCAGCATGTAGGTAGACTGGAGTTCTTTAACAGCAGTGGCTTTTTGTTCTTAACTTGGCATCTATGTTGCTTAGAACATAGTAACTGTTTAA
Sapajus-993.63-KB4207-IntF1        ~~~~~~~~~~~~~~~~~~~~~~~~~~~~~~~~~~~~~~~~~~~~~~~~~~~~~~~~~~~~~~~~~~~~~~~~~~~~~~~~~~~~~~~~~~~~~~~~~~~~
Sapajus-993.63-KB4207-R-rc         ~~~~~~~~~~~~~~~~~~~~~~~~~~~~~~~~~~~~~~~~~~~~~~~~~~~~~~~~~~~~~~~~~~~~~~~~~~~~~~~~~~~~~~~~~~~~~~~~~~~~

                                           610       620       630       640       650       660       670       680       690       700
                                   ....|....|....|....|....|....|....|....|....|....|....|....|....|....|....|....|....|....|....|....|
Sapajus-993.63-S. apella genome    ggccgggcgcggtggctcacgcctgtaatcccagcactttgggaggctgaggcgggtggatcacgaggtcaagagatcgagaccatcctggtcaacatgg
Sapajus-933.63-C. albifrons_rc     ~~~~~~~~~~~~~~~~~~~~~~~~~~~~~~~~~~~~~~~~~~~~~~~~~~~~~~~~~~~~~~~~~~~~~~~~~~~~~~~~~~~~~~~~~~~~~~~~~~~~
Sapajus-993.63-KB4207-F            TTAGTGTTTAATAATGAATTGAATCAAGTGTTTCGTTATCAGTGTTTAATTATGAAATGAATCAATGAGGCCGGGCGCGGTGGCTCAAGCCTGTAATCCC
Sapajus-993.63-KB4207-SIntR1rc     TTAGTGTTTAATAATGAATTGAATCAAGTGTTTCGTTATCAGTGTTTAATTATGAAATGAATCAATGAGGCCGGGCGCGGTGGCTCAAGCCTGTAATCCC
Sapajus-993.63-KB4207-IntF1        ~~~~~~~~~~~~~~~~~~~~~~~~~~~~~~~~~~~~~~~~~~~~~~~~~~~~~~~~~~~~~~~~~~~~~~~~~~~~~~~~~~~~~~~~~~~~~~~~~~~~
Sapajus-993.63-KB4207-R-rc         ~~~~~~~~~~~~~~~~~~~~~~~~~~~~~~~~~~~~~~~~~~~~~~~~~~~~~~~~~~~~~~~~~~~~~~~~~~~~~~~~~~~~~~~~~~~~~~~~~~~~
```

```
                                          710       720       730       740       750       760       770       780       790       800
                                  ....|....|....|....|....|....|....|....|....|....|....|....|....|....|....|....|....|....|....|....|
Sapajus-993.63-S. apella genome   tgaaaccctgtctctactaaaaatacaaaaaattagctgggcatggtggcacgtgcctgtaatcccagctactcaggaggctgaggcaggagaattgcct
Sapajus-933.63-C. albifrons_rc    ~~~~~~~~~~~~~~~~~~~~~~~~~~~~~~~~~~~~~~~~~~~~~~~~~~~~~~~~~~~~~~~~~~~~~~~~~~~~~~~~~~~~~~~~~~~~~~~~~~~~
Sapajus-993.63-KB4207-F           AGCACTGTGGGAGGCCGAGGCGGGTGGATCACGAGGTCAAGAGATCGAGACCATCCTGGTCAACATGGTGAAACCCCGTCTTTACTAAAAATACAAAAAA
Sapajus-993.63-KB4207-SIntR1rc    AGCACTGTGGGAGGCCGAGGCGGGTGGATCACGAGGTCAAGAGATCGAGACCATCCTGGTCAACATGGTGAAACCCCGTCTTTACTAAAAATACAAAAAA
Sapajus-993.63-KB4207-IntF1       ~~~~~~~~~~~~~~~~~~~~~~~~~~~~~~~~GAGGTCAAGAGATCGAGACCATCCTGGTCAACATGGTGAAACCCCGTCTTTACTAAAAATACAAAAAA
Sapajus-993.63-KB4207-R-rc        ~~~~~~~~~~~~~~~~~~~~~~~~~~~~~~~~~~~~~~~~~~~~~~~~~~~~~~~~~~~~~~~~~~~~~~~~~~~~~~~~~~~~~~~~~~~~~~~~~~~~
                                          810       820       830       840       850       860       870       880       890       900
                                  ....|....|....|....|....|....|....|....|....|....|....|....|....|....|....|....|....|....|....|....|
Sapajus-993.63-S. apella genome   gaacccaggaggcggaggttgcggtgagctgagatcgcgccattgcactccagcctgggtaacaaaagcgaaactccgtctcaaaaaaaaaaaaaaaaaa
Sapajus-933.63-C. albifrons_rc    ~~~~~~~~~~~~~~~~~~~~~~~~~~~~~~~~~~~~~~~~~~~~~~~~~~~~~~~~~~~~~~~~~~~~~~~~~~~~~~~~~~~~~~~~~~~~~~~~~~~~
Sapajus-993.63-KB4207-F           TTAGCTGGGCATCGTGGCGCGTGCCTGTAATCCC
Sapajus-993.63-KB4207-SIntR1rc    TTAGCTGGGCATCGTGGCGCGTGCCTGTAATCCCAGCGACTCAGGAGG
Sapajus-993.63-KB4207-IntF1       TTAGCTGGGCATCGTGGCGCGTGCCTGTAATCCCAGCGACTCAGGAGGCTGGGGCAGGAGAATTGCCTGAACCCGGGAGGCGGAGGTTGCGGTGAGCCGA
Sapajus-993.63-KB4207-R-rc        ~~~~~~~~~~~~~~~~~~~~~~~~~~~~~~~~~~~~~~~~~~~~~~~~~~~~~~~~~~~~~~~~~~~~~~~~~~~~~~~~~~~~~~~~~~~~~~~~~~~~
                                          910       920       930       940       950       960       970       980       990      1000
                                  ....|....|....|....|....|....|....|....|....|....|....|....|....|....|....|....|....|....|....|....|
Sapajus-993.63-S. apella genome   gaacatagtaACTATTTAATTAGTGTTTAATAATGAAGTGAATCAAGTGTTTCGTTATGTGTTT~~AATTATGAAATGAATCAATGATCATGTTCCCTag
Sapajus-933.63-C. albifrons_rc    ~~~~~~~~~~~~~~~~~~~TTAGTGTTTAATAATGAATTGAATCAAGTGTTTCGTTATCAGTGTTTAATTATGAAATGAATCAATGATCATGTTCCCTAG
Sapajus-993.63-KB4207-F
Sapajus-993.63-KB4207-SIntR1rc
Sapajus-993.63-KB4207-IntF1       GATCGCGCCATTGCACTCCAGCCTGGGTAACAAGAGCGAAACTCCGTCTCAAAA
Sapajus-993.63-KB4207-R-rc        ~~~~~~~~~~~~~~~~~~~~~~~~~~~~~~~~~~~~~~~~~~~~AAAAAAAAAAAAAAAAAAAAAAAAAAAAGAAATGAATCAATGATCATGTTCCCTAG
```

```
                                          310       320       330       340       350       360       370       380       390       400
                                  ....|....|....|....|....|....|....|....|....|....|....|....|....|....|....|....|....|....|....|....|
Sapajus-942.22-S. apella_rc       TATGTGTGCCTGAAATAATCAAGGCCTCGCTCATTTATTCAGTATTTGAATATCTCCTGGGCTTCAGACTTTGTGCCCAGAAGTAGGATACAGCAAAGAA
Sapajus-942.22-C. imitator_rc     TATGTGTGCCTGAAATAATCAAGGCCTCGCTCATTTATTCAGTATTTGAATATCTCCTGGGCTTCAGACTTTGTGCCCAGAAGTAGGATACAGCAAAGAA
Sapajus-942.22-C. albifrons_rc    ~~~~~~~~~~~~~~~~~~~~AAGGCCTCGCTCATTTATTCAGTATTTGAATATCTCCTGGGCTTCAGACTTTGTGCCCAGAAGTAGGATACAGCAAAGAA
Sapajus-942.22-Cc_AM_T3-R         ~~~~~~~~~~~~~~~~~~~~~~~~~~~~~~~~~~~~~~~~~~~~~~~~~~~~~~~~~~~~~~~~~~~~~~~~~~~~~~~~~~~~~~~~~~~~~~~~~~~~
Sapajus-942.22-Cc_AM_T3-SIntR1rc  ~~~~~~~~~~~~~~~~~~~~~~~~~~~~~~~~~~~~~~~~~~~~~~~~~~~~~~~~~~~~~~~~~~~~~~~~~~~~~~~~~~~~~~~~~~~~~~~~~~~~
Sapajus-942.22-Cc_AM_T3-IntF1     ~~~~~~~~~~~~~~~~~~~~~~~~~~~~~~~~~~~~~~~~~~~~~~~~~~~~~~~~~~~~~~~~~~~~~~~~~~~~~~~~~~~~~~~~~~~~~~~~~~~~
Sapajus-942.22-Cc_AM_T3-F-rc      ~~~~~~~~~~~~~~~~~~~~~~~~~~~~~~~~~~~~~~~~~~~~~~~~~~~~~~~~~~~~~~~~~~~~~~~~~~~~~~~~~~~~~~~~~~~~~~~~~~~~
                                          410       420       430       440       450       460       470       480       490       500
                                  ....|....|....|....|....|....|....|....|....|....|....|....|....|....|....|....|....|....|....|....|
Sapajus-942.22-S. apella_rc       CAGACCTGTTCTCTGTCTTAAGTGAGGGAGCTCACAATTTGGTAGTGCAACTGCCCAAATGTTTTTCAGTAAGTGCTAGTAGATGTCTTTATCAATTGCC
Sapajus-942.22-C. imitator_rc     CAGACCTGTTCTCTGTCTTAAGTGAGGGAGCTCACAATTTGGTAGTGCAACTGCCCAAATGTTTTTCAGTAAGTGCTAGTAGATGTCTTTATCAATTGCC
Sapajus-942.22-C. albifrons_rc    CAGACCTGTTCTCTGTCTTAAGTGAGGGAGCTCACAATTTGGTAGTGCAACTGCCCAAATGTTTTTCAGTAAGTGCTAGTAGATGTCTTTATCAATTGCC
Sapajus-942.22-Cc_AM_T3-R         ~~~~~~~~~~~~~~~~~~~~~~~~~~~~~~~~~~~~~~~~~~~~~~~~~~~~~~~~~~~~~~~~~~~~~~~~~~~~~~~~~~~~~~~~~~~~~~~~~~~~
Sapajus-942.22-Cc_AM_T3-SIntR1rc  ~~~~~~~~~~~~~~~~~~~~~~~~~~~~~~~~~~~~~~~~~~~~~~~~~~~~~~~~~~~~~~~~~~~~~~~~~~~~~~~~~~~~~~~~~~~~~~~~~~~~
Sapajus-942.22-Cc_AM_T3-IntF1     ~~~~~~~~~~~~~~~~~~~~~~~~~~~~~~~~~~~~~~~~~~~~~~~~~~~~~~~~~~~~~~~~~~~~~~~~~~~~~~~~~~~~~~~~~~~~~~~~~~~~
Sapajus-942.22-Cc_AM_T3-F-rc      ~~~~~~~~~~~~~~~~~~~~~~~~~~~~~~~~~~~~~~~~~~~~~~~~~~~~~~~~~~~~~~~~~~~~~~~~~~~~~~~~~~~~~~~~~~~~~~~~~~~~
                                          510       520       530       540       550       560       570       580       590       600
                                  ....|....|....|....|....|....|....|....|....|....|....|....|....|....|....|....|....|....|....|....|
Sapajus-942.22-S. apella_rc       ACAGGTGAGGGGAATCGAGGGAGGTCTTTCCAAAGGACAGAACATCTGAGCACTGATTTTCAAAGAACGTGTCTGTCAAGCAGATAAGAGGGCagaagag
Sapajus-942.22-C. imitator_rc     ACAGGTGAGGGGAATCGAGGGAGGTCTTTCCAAAGGAAAGAACATCTGAGCACTGATTTTCAAAGAACGTGTCTGTCAAGCAGATAAGAGGGCAGAAGAG
Sapajus-942.22-C. albifrons_rc    AGAGGTGAGGGGAATCGAGGGAGGTCTTTCCAAAGGAAAGAACATCTGAGCACTGATTTTCAAAGAACGTGTCTGTCAAGCAGATAAGAGGGCAGAAGAG
Sapajus-942.22-Cc_AM_T3-R         ~~~~~~~~~~~~~~~~~~~~~~~~~~~~~~~~~~~~~~~~~~~~~~~~~~~~~~~~~~~~~~~~~~~~~~~~~~~~~~~~~~~~~~~~~~~~~~~~~~~~
Sapajus-942.22-Cc_AM_T3-SIntR1rc  ~~~~~~~~~~~~~~~~~~~~~~~~~~~~~~~~~~~~~~~~~~~~~~~~~~~~~~~~~~~~~~~~~~~~~~~~~~~~~~~~~~~~~~~~AGGGCAGAAGAG
Sapajus-942.22-Cc_AM_T3-IntF1     ~~~~~~~~~~~~~~~~~~~~~~~~~~~~~~~~~~~~~~~~~~~~~~~~~~~~~~~~~~~~~~~~~~~~~~~~~~~~~~~~~~~~~~~~~~~~~~~~~~~~
Sapajus-942.22-Cc_AM_T3-F-rc      ~~~~~~~~~~~~~~~~~~~~~~~~~~~~~~~~~~~~~~~~~~~~~~~~~~~~~~~~~~~~~~~~~~~~~~~~~~~~~~~~~~~~~~~~~~~~~~~~~~~~
```

```
                                                610       620       630       640       650       660       670       680       690       700
                                       ....|....|....|....|....|....|....|....|....|....|....|....|....|....|....|....|....|....|....|....|
Sapajus-942.22-S. apella_rc            ccgggcgcggtggctcacgcctgtaatcccagcactttgggaggccgaggcgggtggatcacgaggtcaagagatcgagaccatcctggtcaacatggtg
Sapajus-942.22-C. imitator_rc          ~~~~~~~~~~~~~~~~~~~~~~~~~~~~~~~~~~~~~~~~~~~~~~~~~~~~~~~~~~~~~~~~~~~~~~~~~~~~~~~~~~~~~~~~~~~~~~~~~~~~
Sapajus-942.22-C. albifrons_rc         CCGGGCACGGTGGCTCATGCTTGTAATCCCAGCACTTTGGGAGGCCGAGGCGGGTGGATCACGAGGTCAAGAGATCGAGACCATCCTGGTCAACATGGTG
Sapajus-942.22-Cc_AM_T3-R              ~~~~~~~~~~~~~~~~~~~~~~~~~~~~~~~~~~~~~~~~~~~~~~~~~~~~GGTGGATCACGAGGTCAAGAGATCGAGACCATCCTGGTCAACATGGTG
Sapajus-942.22-Cc_AM_T3-SIntR1rcCCGGGCACGGTGGCTCACGCTTGTAATCCCAGCACTTTGGGAGGCCGAGGCGGGTGGATCACGAGGTCAAGAGATCGAGACCATCCTGGTCAACATGGTG
Sapajus-942.22-Cc_AM_T3-IntF1          ~~~~~~~~~~~~~~~~~~~~~~~~~~~~~~~~~~~~~~~~~~~~~~~~~~~~~~~~~~~~~~GAGGTCAAGAGATCGAGACCATCCTGGTCAACATGGTG
Sapajus-942.22-Cc_AM_T3-F-rc           ~~~~~~~~~~~~~~~~~~~~~~~~~~~~~~~~~~~~~~~~~~~~~~~~~~~~~~~~~~~~~~~~~~~~~~~~~~~~~~~~~~~~~~~~~~~~~~~~~~~
                                                710       720       730       740       750       760       770       780       790       800
                                       ....|....|....|....|....|....|....|....|....|....|....|....|....|....|....|....|....|....|....|....|
Sapajus-942.22-S. apella_rc            aaaccccgtctctactaaaactacaaaacattagctgggcatggtggcgcgtgcctgtaatcccagctacccaggaggctgaggcaggagaattgcctga
Sapajus-942.22-C. imitator_rc          ~~~~~~~~~~~~~~~~~~~~~~~~~~~~~~~~~~~~~~~~~~~~~~~~~~~~~~~~~~~~~~~~~~~~~~~~~~~~~~~~~~~~~~~~~~~~~~~~~~~~
Sapajus-942.22-C. albifrons_rc         AAACCCCGTCTCTACTAAAAATACAAAACATTAGCTGGGCATGGTGGCGCGTGCCTGTAGTCCCAGCTACTCAGGAGGCTGAGGCAGGAGAATTGCCTGA
Sapajus-942.22-Cc_AM_T3-R              AAACCCCGTCTCTACTAAAAATACAA
Sapajus-942.22-Cc_AM_T3-SIntR1rcAAACCCCGTCTCTACTAAAAATACAAAACATTAGCTGGGCATGGTGGCGCGTGCCTGTAGTCCCAGCTACTCAGGAGG
Sapajus-942.22-Cc_AM_T3-IntF1          AAACCCCGTCTCTACTAAAAATACAAAACATTAGCTGGGCATGGTGGCGCGTGCCTGTAGTCCCAGCTACTCAGGAGGCTGAGGCAGGAGAATTGCCTGA
Sapajus-942.22-Cc_AM_T3-F-rc           ~~~~~~~~~~~~~~~~~~~~~~~~~~~~~~~~~~~~~~~~~~~~~~~~~~~~~~~~~~~~~~~~~~~~~~~~~~~~~~~~~~~~~~~~~~~~~~~~~~~
                                                810       820       830       840       850       860       870       880       890       900
                                       ....|....|....|....|....|....|....|....|....|....|....|....|....|....|....|....|....|....|....|....|
Sapajus-942.22-S. apella_rc            acccaggaggcggaggttgtggtgagccgagatcgtgccattgcactccagcctgggtaacaagagcgaaactctgtctcaaaaaaaaaaaaaaaaaaaa
Sapajus-942.22-C. imitator_rc          ~~~~~~~~~~~~~~~~~~~~~~~~~~~~~~~~~~~~~~~~~~~~~~~~~~~~~~~~~~~~~~~~~~~~~~~~~~~~~~~~~~~~~~~~~~~~~~~~~~~
Sapajus-942.22-C. albifrons_rc         ACCCAGGAGGCGGAGGTTGC
Sapajus-942.22-Cc_AM_T3-R
Sapajus-942.22-Cc_AM_T3-SIntR1rc
Sapajus-942.22-Cc_AM_T3-IntF1          ACCCAGGAGGCGGAGGTTGCGGTGAGCCGAGATCGCGCCATTGCACTCCAGCCTGGGTAACAAGAGCGAAACTCCGTCTCAAAAAAAA
Sapajus-942.22-Cc_AM_T3-F-rc           ~~~~~~~~~~~~~~~~~~~~~~~~~~~~~~~~~~~~~~~~~~~~~~~~~~~~~~~~~~~~~~~~~~~~~~~~~~~~~~~~~~~~~~~~~~~~~~

                                                910       920       930       940       950       960       970       980       990      1000
                                       ....|....|....|....|....|....|....|....|....|....|....|....|....|....|....|....|....|....|....|....|
Sapajus-942.22-S. apella_rc            aaaaaaaaaaaaaaaaaaggcagaagagaaGTCCAGGTGAAAGCAAGGTGCAGGTAAGTCCAGGCAGAGTGAGAGAGCTCATGAAGAGCTTGGGGACCCT
Sapajus-942.22-C. imitator_rc          ~~~~~~~~~~~~~~~~~~~~~~~~~~~~AAGTCCAGGTGAAAGCAAGGTGCAGGTAAGTCCAGGCAGAGTGAGAGAGCTCACGAAGAGCTTGGGGACCCT
Sapajus-942.22-C. albifrons_rc
Sapajus-942.22-Cc_AM_T3-R
Sapajus-942.22-Cc_AM_T3-SIntR1rc
Sapajus-942.22-Cc_AM_T3-IntF1
Sapajus-942.22-Cc_AM_T3-F-rc           AAAAAAAAAAAAAAAGAGGGCAGAAGAGAAGTCCAGGTGAAAGCAAGGTGCAGGTAAGTCCAGGCAGAGTGAGAGAGCTCACGAAGAGCTTGGGGACCCT
```

```
                                         10        20        30        40        50        60        70        80        90       100
                                 ....|....|....|....|....|....|....|....|....|....|....|....|....|....|....|....|....|....|....|....|
Sapajus-954.16-S. apella genome  ~~~~~~~~~~~~~~~~~~~~~~~~~tgtgtgtgtgtgtgtgtgtgtgtgtgtgtgtgttgagtgtaCGTGCTGCTGTCTTCTCCTTTCTGGAGGCTGTCC
Sapajus-954.16-C. imitator geno  ~~~~~~~~~~~~~~~~~~~~~~~~~~~~~~~~~~~~~~~~~~~~~~~~~~~~~~~~~~~~~~~~~~CGTGCTGCTGTCTTCTCCTTTCTGGAGGCTGTCC
Sapajus-954.16-Cc_AM_T3-F        ~~~~~~~~~~~~~~~~~~~~~~~~~~~~~~~~~~~~~~~~~~~~~~~~~~~~~~~~~~~~~~~~~~~~~~~~~~~~~~~~~~~~~~~~~~~~~~~~~~~~
Sapajus-954.16-Cc_AM_T3-R_rc     ~~~~~~~~~~~~~~~~~~~~~~~~~~~~~~~~~~~~~~~~~~~~~~~~~~~~~~~~~~~~~~~~~~~~~~~~~~~~~~~~~~~~~~~~~~~~~~~~~~~~
Sapajus-954.16-Cc_AM_T3-SIntR1   ~~~~~~~~~~~~~~~~~~~~~~~~~~~~~~~~~~~~~~~~~~~~~~~~~~~~~~~~~~~~~~~~~~~~~~~~~~~~~~~~~~~~~~~~~~~~~~~~~~~~
Sapajus-954.16-Cc_AM_T3-IntF1_rcTTTTTTTGAGACGGAGTTTCACTCATGTTACCCAGGCTGGAGTGCAATGGCGCGATCTCGGCTCACCGCAACCTCCGCCTCCTGGGTTCAGGCAATTCTC

                                        110       120       130       140       150       160       170       180       190       200
                                 ....|....|....|....|....|....|....|....|....|....|....|....|....|....|....|....|....|....|....|....|
Sapajus-954.16-S. apella genome  TGTCAAAGGCCTTTCAGATAGGCAACCAAGGCACATGAACATGTGTTCTCAGATCCAGGCTACTTAACCATTGGTTTGCATAAATGGAGTGGACCCTGtc
Sapajus-954.16-C. imitator geno  TGTCAAAGGCCTTCCAGATAGGCACCCAAGGCACATGAACATGTGTTCTCAGATCCAGGCTACTTAACCATTGGTTTGCATAAATGGAGTGGACCCTGTC
Sapajus-954.16-Cc_AM_T3-F        ~~~~~~~~~~~~~~~~~~~~~~~~~~~~~GGCACATGAACATGTGTTCTCAGATCCAGGCTACTTAACCATTGGTTTGCATAAATGGAGTGGACCCTGTC
Sapajus-954.16-Cc_AM_T3-R_rc     ~~~~~~~~~~~~~~~~~~~~~~~~~~~~~~~~~~~~~~~~~~~~~~~~~~~~~~~~~ATTTTTTTGTGTTTTTAGTAGAGACGGGGTTTCACCATGTTGA
Sapajus-954.16-Cc_AM_T3-SIntR1   ~~~~~~~~~~~CCTCCTGAGTAGCTGGGATTACAGGCACGTGCCACCATGCCCAGCTAATTTTTTGTGTTTTTAGTAGAGACGGGGTTTCACCATGTTGA
Sapajus-954.16-Cc_AM_T3-IntF1rc  CTGCCTCAGCCTCCTGAGTAGCTGGGATTACAGGCANGTNNNNGACATNNNTTAGNTAATTTTTTGTGTTTTTAGTAGAGACGGGGTTTCACCATGTTGA

                                        210       220       230       240       250       260       270       280       290       300
                                 ....|....|....|....|....|....|....|....|....|....|....|....|....|....|....|....|....|....|....|....|
Sapajus-954.16-S. apella genome  atccctccctgccttcctccctccccaccttcatCCCTCATCAGGTTAgtcagaaaaattattattattgtaccaCTCTAAGTTCATTTTACGAATATTA
Sapajus-954.16-C. imitator geno  ATCCCTCCCTGCCTTCCTCCCTCCCCACCTTCATCCCTCATCAGGTTAGTCAGAAAAATTATTATTATTGTACCACTCTAAGTTCATTTTATGAATATTA
Sapajus-954.16-Cc_AM_T3-F        ATCCCTCCCTGCCTTCCTCCCTCCCCACCTTCATCCCTCATCAGGTTAGTCAGAAAAATTATTATTATTGTACCACTCTAAGTTCATTTTATGAATATTA
Sapajus-954.16-Cc_AM_T3-R_rc     CCAGGATGGTCTCGATCTCTTGACCTCGTGATCCACCCGCCTCGGCCTCCCAAAGTGCTGGGATTACAGGCTTGAGCCACCGCGCCCGGCATGAATATTA
Sapajus-954.16-Cc_AM_T3-SIntR1   CCAGGATGGTCTCGATCTCTTGACCTCGTGATCCACCCGCCTCGGCCTCCCAAAGTGCTGGGATTACAGGCTTGAGCCACCGCGCCCGGCATGAATATTA
Sapajus-954.16-Cc_AM_T3-IntF1rc  CCAGGATGGTCTCGATCTCTTGACC

                                        310       320       330       340       350       360       370       380       390       400
                                 ....|....|....|....|....|....|....|....|....|....|....|....|....|....|....|....|....|....|....|....|
Sapajus-954.16-S. apella genome  CTTTCTAAACCACActctcatttgttttctctttttttccccttctgtcaaCCCTAGGGGCAGGTAAAGGCAGATAAAGTTCatattttaaggccgggcg
Sapajus-954.16-C. imitator geno  CTTTCTAAACCACACTCTCATTTGTTTTCTCTTTTTTTCCCCTTCTGTCAACCCTAGGGGCAGGTAAAGGCAGATAAAGTTCATATTTTA~~~~~~~~~~
Sapajus-954.16-Cc_AM_T3-F        CTTTCT
Sapajus-954.16-Cc_AM_T3-R_rc     CTTTCTAAACCACACTCTCATTTGTTTTCTCTTTTTTTCCCCTTCTGTCAACCCTAGGGGCAGGTAAAGGCAGATAAAGTTCATATTTTA~~~~~~~~~~
Sapajus-954.16-Cc_AM_T3-SIntR1   CTTTCTAAACCACACTCTCATTTGTTTTCTCTTTTTTTCCCCTTCTGTCAACCCTAGGGGCAGGTAAAGGCAGATAAAGTTCATATTTTA~~~~~~~~~~
Sapajus-954.16-Cc_AM_T3-IntF1rc

                                        410       420       430       440       450       460       470       480       490       500
                                 ....|....|....|....|....|....|....|....|....|....|....|....|....|....|....|....|....|....|....|....|
Sapajus-954.16-S. apella genome  cggtggctcaagcctgtaatcccagcactttgggaggccgaggcgggtggatcacgaggtcaagagatcgagaccatcctggtcaacatggtgaaacccc
Sapajus-954.16-C. imitator geno  ~~~~~~~~~~~~~~~~~~~~~~~~~~~~~~~~~~~~~~~~~~~~~~~~~~~~~~~~~~~~~~~~~~~~~~~~~~~~~~~~~~~~~~~~~~~~~~~~~~~~
Sapajus-954.16-Cc_AM_T3-F
Sapajus-954.16-Cc_AM_T3-R_rc     ~~~~~~~~~~~~~~~~~~~~~~~~~~~~~~~~~~~~~~~~~~~~~~~~~~~~~~~~~~~~~~~~~~~~~~~~~~~~~~~~~~~~~~~~~~~~~~~~~~~~
Sapajus-954.16-Cc_AM_T3-SIntR1   ~~~~~~~~~~~~~~~~~~~~~~~~~~~~~~~~~~~~~~~~~~~~~~~~~~~~~~~~~~~~~~~~~~~~~~~~~~~~~~~~~~~~~~~~~~~~~~~~~~~~
Sapajus-954.16-Cc_AM_T3-IntF1rc
```

```
                                                   510       520       530       540       550       560       570       580       590       600
                                           ....|....|....|....|....|....|....|....|....|....|....|....|....|....|....|....|....|....|....|....|
Sapajus-954.16-S. apella genome            gtctctactaaaaaatacaaaaaaaaaaaaattagctgggcatggtggcgcgtgcctgtaatcccagctactcaggaggctgaggcaggagaattgcctg
Sapajus-954.16-C. imitator geno            ~~~~~~~~~~~~~~~~~~~~~~~~~~~~~~~~~~~~~~~~~~~~~~~~~~~~~~~~~~~~~~~~~~~~~~~~~~~~~~~~~~~~~~~~~~~~~~~~~~~~
Sapajus-954.16-Cc_AM_T3-F
Sapajus-954.16-Cc_AM_T3-R_rc               ~~~~~~~~~~~~~~~~~~~~~~~~~~~~~~~~~~~~~~~~~~~~~~~~~~~~~~~~~~~~~~~~~~~~~~~~~~~~~~~~~~~~~~~~~~~~~~~~~~~~
Sapajus-954.16-Cc_AM_T3-SIntR1             ~~~~~~~~~~~~~~~~~~~~~~~~~~~~~~~~~~~~~~~~~~~~~~~~~~~~~~~~~~~~~~~~~~~~~~~~~~~~~~~~~~~~~~~~~~~~~~~~~~~~
Sapajus-954.16-Cc_AM_T3-IntF1rc

                                                   610       620       630       640       650       660       670       680       690       700
                                           ....|....|....|....|....|....|....|....|....|....|....|....|....|....|....|....|....|....|....|....|
Sapajus-954.16-S. apella genome            aacccaggaggcggaggttgcggtgagccgagatcgcgccattgcactccagcctgggtaacaagagcgaaactccgtctcaaaaaaaaaaaaaaaaaaa
Sapajus-954.16-C. imitator geno            ~~~~~~~~~~~~~~~~~~~~~~~~~~~~~~~~~~~~~~~~~~~~~~~~~~~~~~~~~~~~~~~~~~~~~~~~~~~~~~~~~~~~~~~~~~~~~~~~~~~~
Sapajus-954.16-Cc_AM_T3-F
Sapajus-954.16-Cc_AM_T3-R_rc               ~~~~~~~~~~~~~~~~~~~~~~~~~~~~~~~~~~~~~~~~~~~~~~~~~~~~~~~~~~~~~~~~~~~~~~~~~~~~~~~~~~~~~~~~~~~~~~~~~~~~
Sapajus-954.16-Cc_AM_T3-SIntR1             ~~~~~~~~~~~~~~~~~~~~~~~~~~~~~~~~~~~~~~~~~~~~~~~~~~~~~~~~~~~~~~~~~~~~~~~~~~~~~~~~~~~~~~~~~~~~~~~~~~~~
Sapajus-954.16-Cc_AM_T3-IntF1rc

                                                   710       720       730       740       750       760       770       780       790       800
                                           ....|....|....|....|....|....|....|....|....|....|....|....|....|....|....|....|....|....|....|....|
Sapajus-954.16-S. apella genome            agttcatattttatagattaaaaactAGGGAAGCTAGAAGAGAAATAAACTCCAGTTTATGGAGTTTCAACTTCCTGTTGTTTTCATAGGCCCGGATGAC
Sapajus-954.16-C. imitator geno            ~~~~~~~~~~~~~TAGATTAAAAACTAGGGAAGCTAGAAGAGAAATAAACTCCAGTTTATAGAGTTTCAGCTTCCTGTTGTTTTCATAGGCCCGGATGAC
Sapajus-954.16-Cc_AM_T3-F
Sapajus-954.16-Cc_AM_T3-R_rc               ~~~~~~~~~~~~~TAGATTAAAAACTAGGGAAGCTAGAAGAG
Sapajus-954.16-Cc_AM_T3-SIntR1             ~~~~~~~~~~~~~TAGATTAAAAACTAGGGAAGCTAGAAGAGAAATAAACTCCAGTTTATAGAGTTTCAGCTTCCTGTTGTTTTCATAGGCCCGGATA
Sapajus-954.16-Cc_AM_T3-IntF1rc

                                                    10        20        30        40        50        60        70        80        90       100
                                           ....|....|....|....|....|....|....|....|....|....|....|....|....|....|....|....|....|....|....|....|
Reverse complement
Sapajus-954.16-S. apella genome            CTCTTTGGTCACTCTCTGGCATGTTTTAGAAGCAACTTAATCAGAGATAGGGTCAGGTCAATGGCAGAACGAAAGACATGGGCTTTAGAGTTATTAAGGC
Sapajus-954.16-C. imitator geno            CTCTTTGGTCACTCTCTGACATGTTTTAGAAGCAACTTAATCAGAGATAGGGTCAGGTCAATGGCAGAACGAAAGACATGGGCTTTAGAGTTATTAAGAC
Sapajus-954.16-Cc_AM_T3-SIntR1rc           ~~~~~~~~~~~~~~~~~~~~~~~~~~~~~~~~~~~~~~~~~~~~~~~~~~~~~~~~~~~~~~~~~~~~~~~~~~~~~~~~~~~~~~~~~~~~~~~~~~~~
Sapajus-954.16-Cc_AM_T3-R                  ~~~~~~~~~~~~~~~~~~~~~~~~~~~~~~~~~~~~~~~~~~~~~~~~~~~~~~~~~~~~~~~~~~~~~~~~~~~~~~~~~~~~~~~~~~~~~~~~~~~~
Sapajus-954.16-Cc_AM_T3-IntF1              ~~~~~~~~~~~~~~~~~~~~~~~~~~~~~~~~~~~~~~~~~~~~~~~~~~~~~~~~~~~~~~~~~~~~~~~~~~~~~~~~~~~~~~~~~~~~~~~~~~~~
Sapajus-954.16-Cc_AM_T3-F_rc               ~~~~~~~~~~~~~~~~~~~~~~~~~~~~~~~~~~~~~~~~~~~~~~~~~~~~~~~~~~~~~~~~~~~~~~~~~~~~~~~~~~~~~~~~~~~~~~~~~~~~
Sapajus-954.16-UF31995-SIntR1rc            ~~~~~~~~~~~~~~~~~~~~~~~~~~~~~~~~~~~~~~~~~~~~~~~~~~~~~~~~~~~~~~~~~~~~~~~~~~~~~~~~~~~~~~~~~~~~~~~~~~~~
Sapajus-954.16-UF31995-R                   ~~~~~~~~~~~~~~~~~~~~~~~~~~~~~~~~~~~~~~~~~~~~~~~~~~~~~~~~~~~~~~~~~~~~~~~~~~~~~~~~~~~~~~~~~~~~~~~~~~~~
Sapajus-954.16-UF31995-F_rc                ~~~~~~~~~~~~~~~~~~~~~~~~~~~~~~~~~~~~~~~~~~~~~~~~~~~~~~~~~~~~~~~~~~~~~~~~~~~~~~~~~~~~~~~~~~~~~~~~~~~~
```

```
                                                 110       120       130       140       150       160       170       180       190       200
                                        ....|....|....|....|....|....|....|....|....|....|....|....|....|....|....|....|....|....|....|....|
Reverse complement
Sapajus-954.16-S. apella genome         TTGGGTCCGAGTTCTGATGTCACTGCATAACGATAGCTCTAGATCTCAACTTAGAACAAACACATATCAAATAATTTGGGAGATTAAAAGAGCTGATGAT
Sapajus-954.16-C. imitator geno         TTGGGTCCGAGTTCTGATGTCACTGCATAACGATAGCTCTAGATCTCAACTTAGAACAAACACATATCAAATAATTTGGGAGATTAAAAGAGCTGATGAT
Sapajus-954.16-Cc_AM_T3-SIntR1Rc        ~~~~~~~~~~~~~~~~~~~~~~~~~~~~~~~~~~~~~~~~~~~~~~~~~~~~~~~~~~~~~~~~~~~~~~~~~~~~~~~~~~~~~~~~~~~~~~~~~~~~
Sapajus-954.16-Cc_AM_T3-R               ~~~~~~~~~~~~~~~~~~~~~~~~~~~~~~~~~~~~~~~~~~~~~~~~~~~~~~~~~~~~~~~~~~~~~~~~~~~~~~~~~~~~~~~~~~~~~~~~~~~~
Sapajus-954.16-Cc_AM_T3-IntF1           ~~~~~~~~~~~~~~~~~~~~~~~~~~~~~~~~~~~~~~~~~~~~~~~~~~~~~~~~~~~~~~~~~~~~~~~~~~~~~~~~~~~~~~~~~~~~~~~~~~~~
Sapajus-954.16-Cc_AM_T3-F_rc            ~~~~~~~~~~~~~~~~~~~~~~~~~~~~~~~~~~~~~~~~~~~~~~~~~~~~~~~~~~~~~~~~~~~~~~~~~~~~~~~~~~~~~~~~~~~~~~~~~~~~
Sapajus-954.16-UF31995-SIntR1rc         ~~~~~~~~~~~~~~~~~~~~~~~~~~~~~~~~~~~~~~~~~~~~~~~~~~~~~~~~~~~~~~~~~~~~~~~~~~~~~~~~~~~~~~~~~~~~~~~~~~~~
Sapajus-954.16-UF31995-R                ~~~~~~~~~~~~~~~~~~~~~~~~~~~~~~~~~~~~~~~~~~~~~~~~~~~~~~~~~~~~~~~~~~~~~~~~~~~~~~~~~~~~~~~~~~~~~~~~~~~~
Sapajus-954.16-UF31995-F_rc             ~~~~~~~~~~~~~~~~~~~~~~~~~~~~~~~~~~~~~~~~~~~~~~~~~~~~~~~~~~~~~~~~~~~~~~~~~~~~~~~~~~~~~~~~~~~~~~~~~~~~

                                                 210       220       230       240       250       260       270       280       290       300
                                        ....|....|....|....|....|....|....|....|....|....|....|....|....|....|....|....|....|....|....|....|
Reverse compliment
Sapajus-954.16-S. apella genome         AAAATGCCTGCCATGGTGCTATTACTGTAACCAGGAACCTGCATCTGAGTCTTCACAAAAGGTCATCCGGGCCTATGAAAACAACAGGAAGTTGAAACTC
Sapajus-954.16-C. imitator geno         AAAATGCCTGCCATGGTGCTATTACTGCAACCAGGAACCTGCATCTGAGTCTTCACAAAAGGTCATCCGGGCCTATGAAAACAACAGGAAGCTGAAACTC
Sapajus-954.16-Cc_AM_T3-SIntR1rc        ~~~~~~~~~~~~~~~~~~~~~~~~~~~~~~~~~~~~~~~~~~~~~~~~~~~~~~~~~~~~~~~TATCCGGGCCTATGAAAACAACAGGAAGCTGAAACTC
Sapajus-954.16-Cc_AM_T3-R               ~~~~~~~~~~~~~~~~~~~~~~~~~~~~~~~~~~~~~~~~~~~~~~~~~~~~~~~~~~~~~~~~~~~~~~~~~~~~~~~~~~~~~~~~~~~~~~~~~~~~
Sapajus-954.16-Cc_AM_T3-IntF1           ~~~~~~~~~~~~~~~~~~~~~~~~~~~~~~~~~~~~~~~~~~~~~~~~~~~~~~~~~~~~~~~~~~~~~~~~~~~~~~~~~~~~~~~~~~~~~~~~~~~~
Sapajus-954.16-Cc_AM_T3-F_rc            ~~~~~~~~~~~~~~~~~~~~~~~~~~~~~~~~~~~~~~~~~~~~~~~~~~~~~~~~~~~~~~~~~~~~~~~~~~~~~~~~~~~~~~~~~~~~~~~~~~~~
Sapajus-954.16-UF31995-SIntR1rc         ~~~~~~~~~~~~~~~~~~~~~~~~~~~~~~~~~~~~~~~~~~~~~~~~~~~~~~~~~~~~~~~~~~~~~~~~~~~~~~~~~~~~~~~~~~~~~~~~~~~~
Sapajus-954.16-UF31995-R                ~~~~~~~~~~~~~~~~~~~~~~~~~~~~~~~~~~~~~~~~~~~~~~~~~~~~~~~~~~~~~~~~~~~~~~~~~~~~~~~~~~~~~~~~~~~~~~~~~~~~
Sapajus-954.16-UF31995-F_rc             ~~~~~~~~~~~~~~~~~~~~~~~~~~~~~~~~~~~~~~~~~~~~~~~~~~~~~~~~~~~~~~~~~~~~~~~~~~~~~~~~~~~~~~~~~~~~~~~~~~~~

                                                 310       320       330       340       350       360       370       380       390       400
                                        ....|....|....|....|....|....|....|....|....|....|....|....|....|....|....|....|....|....|....|....|
Reverse complement
Sapajus-954.16-S. apella genome         CATAAACTGGAGTTTATTTCTCTTCTAGCTTCCCTagttttaatctataaaatatgaacttttttttttttttttttttgagacggagtttcgctcttg
Sapajus-954.16-C. imitator geno         TATAAACTGGAGTTTATTTCTCTTCTAGCTTCCCTAGTTTTTAATCTATAAAATATGAACTTTATCTGCCTTTACCTGCCCCTAGGGTTGACAGAAGGGG
Sapajus-954.16-Cc_AM_T3-SIntR1rc        TATAAACTGGAGTTTATTTCTCTTCTAGCTTCCCTAGTTTTTAATCTATAAAATATGAACTTTATCTGCCTTTACCTGCCCCTAGGGTTGACAGAAGGGG
Sapajus-954.16-Cc_AM_T3-R               ~~~~~~~~~~~~~~~~~~~CTCTTCTAGCTTCCCTAGTTTTTAATCTATAAAATATGAACTTTATCTGCCTTTACCTGCCCCTAGGGTTGACAGAAGGGG
Sapajus-954.16-Cc_AM_T3-IntF1           ~~~~~~~~~~~~~~~~~~~~~~~~~~~~~~~~~~~~~~~~~~~~~~~~~~~~~~~~~~~~~~~~~~~~~~~~~~~~~~~~~~~~~~~~~~~~~~~~~~~~
Sapajus-954.16-Cc_AM_T3-F_rc            ~~~~~~~~~~~~~~~~~~~~~~~~~~~~~~~~~~~~~~~~~~~~~~~~~~~~~~~~~~~~~~~~~~~~~~~~~~~~~~~~~~~~~~~~~~~~~~~~~~~~
Sapajus-954.16-UF31995-SIntR1rc         ~~~~~~~~~~~~~~~~~~~~~~~~~~~~~TTCCCTAGTTTTTAATCTATAAAATATGAACTTTATCTGCCTTTACCTGCCCCTAGGGTTGACAGAAGGGG
Sapajus-954.16-UF31995-R                ~~~~~~~~~~~~~~~~~TTCTCTTCTAGCTTCCCTAGTTTTTAATCTATAAAATATGAACTTTATCTGCCTTTACCTGCCCCTAGGGTAGACAGAAGGGG
Sapajus-954.16-UF31995-F_rc             ~~~~~~~~~~~~~~~~~~~~~~~~~~~~~~~~~~~~~~~~~~~~~~~~~~~~~~~~~~~~~~~~~~~~~~~~~~~~~~~~~~~~~~~~~~~~~~~~~~~~

                                                 410       420       430       440       450       460       470       480       490       500
                                        ....|....|....|....|....|....|....|....|....|....|....|....|....|....|....|....|....|....|....|....|
Reverse complement
Sapajus-954.16-S. apella genome         ttacccaggctggagtgcaatggcgcgatctcggctcaccgcaacctccgcctcctgggttcaggcaattctcctgcctcagcctcctgagtagctggga
Sapajus-954.16-C. imitator geno         AAAAAAAGAGAAAACAAATGAGAGTGTGGTTTAGAAAGTAATATTCAT~~~~~~~~~~~~~~~~~~~~~~~~~~~~~~~~~~~~~~~~~~~~~~~~~~~~
Sapajus-954.16-Cc_AM_T3-SIntR1rc        AAAAAAAGAGAAAACAAATGAGAGTGTGGTTTAGAAAGTAATATTCATGCCGGGCGCGGTGGCTCAAGCCTGTAATCCCAGCACTTTGGGAGGCCGAGGC
Sapajus-954.16-Cc_AM_T3-R               AAAAAAAGAGAAAACAAATGAGAGTGTGGTTTAGAAAGTAATATTCATGCCGGGCGCGGTGGCTCAAGCCTGTAATCCCAGCACTTTGGGAGGCCGAGGC
Sapajus-954.16-Cc_AM_T3-IntF1           ~~~~~~~~~~~~~~~~~~~~~~~~~~~~~~~~~~~~~~~~~~~~~~~~~~~~~~~~~~~~~~~~~~~~~~~~~~~~~~~~~~~~~~~~~~~~~~~~~~~~
Sapajus-954.16-Cc_AM_T3-F_rc            ~~~~~~~~~~~~~~~~~~~~~~~~~~~~~~~~~~~~~~~~~~~~~~~~~~~~~~~~~~~~~~~~~~~~~~~~~~~~~~~~~~~~~~~~~~~~~~~~~~~~
Sapajus-954.16-UF31995-SIntR1rc         AAAAAAAGAGAAAACAAATGAGAGTGTGGTTTAGAAAGTAATATTCATGCCGGGCGCGGTGGCTCAAGCCTGTAATCCCAGCACTTTGGGAGGCCGAGGC
Sapajus-954.16-UF31995-R                AAAAAAAGAGAAAACAAATGAGAGTGTGGTT
Sapajus-954.16-UF31995-F_rc             ~~~~~~~~~~~~~~~~~~~~~~~~~~~~~~~~~~~~~~~~~~~~~~~~~~~~~~~~~~~~~~~~~~~~~~~~~~~~~~~~~~~~~~~~~~~~~~~~~~~~
```

```
                                           510       520       530       540       550       560       570       580       590       600
                                  ....|....|....|....|....|....|....|....|....|....|....|....|....|....|....|....|....|....|....|....|
Reverse Complement
Sapajus-954.16-S. apella genome   ttacaggcacgcgccaccatgcccagctaattttttttttttttgtattttttagtagagacggggtttcaccatgttgaccaggatggtctcgatctctt
Sapajus-954.16-C. imitator geno   ~~~~~~~~~~~~~~~~~~~~~~~~~~~~~~~~~~~~~~~~~~~~~~~~~~~~~~~~~~~~~~~~~~~~~~~~~~~~~~~~~~~~~~~~~~~~~~~~~~~~
Sapajus-954.16-Cc_AM_T3-SIntR1rcGGGTGGATCACGAGGTCAAGAGATCGAGACCATCCTGGTCAACATGGTGAAACCCCGTCTCTACTAAAAACACAAAAAATTAGCTGGGCATGGTGGCACG
Sapajus-954.16-Cc_AM_T3-R         GGGTGGATCACGAGGTCAAGAGATCGAGACCATCCTGGTCAACATGGTGAAACCCCGTCTCTACTAAAAACACAAAAAAAT
Sapajus-954.16-Cc_AM_T3-IntF1     ~~~~~~~~~~~~~~~~~~~~~~~~~~~GACCATCCTGGTCAACATGGTGAAACCCCGTCTCTACTAAAAACACAAAAAATTANCTAANNNATGTCNNNNA
Sapajus-954.16-Cc_AM_T3-F_rc      ~~~~~~~~~~~~~~~~~~~~~~~~~~~~~~~~~~~~~~~~~~~~~~~~~~~~~~~~~~~~~~~~~~~~~~~~~~~~~~~~~~~~~~~~~~~~~~~~~~~~
Sapajus-954.16-UF31995-SIntR1rc   GGGTGGATCACGAGGTCAAGAGATCGAGACCATCCTGGTCAACATGGTGAAACCCCGTCTCTACTAAAAACACAAAAAATTAGCTGGGCATGGTGGCACG
Sapajus-954.16-UF31995-R
Sapajus-954.16-UF31995-F_rc       ~~~~~~~~~~~~~~~~~~~~~~~~~~~~~~~~~~~~~~~~~~~~~~~~~~~~~~~~~~~~~~~~~~~~~~~~~~~~~~~~~~~~~~~~~~~~~~~~~~~~

                                           610       620       630       640       650       660       670       680       690       700
                                  ....|....|....|....|....|....|....|....|....|....|....|....|....|....|....|....|....|....|....|
Reverse Complement
Sapajus-954.16-S. apella genome   gacctcgtgatccacccgcctcggcctcccaaagtgctgggattacaggcttgagccaccgcgcccggccttaaaatatGAACTTTATCTGCCTTTACCT
Sapajus-954.16-C. imitator geno   ~~~~~~~~~~~~~~~~~~~~~~~~~~~~~~~~~~~~~~~~~~~~~~~~~~~~~~~~~~~~~~~~~~~~~~~~~~~~~~~~~~~~~~~~~~~~~~~~~~~~
Sapajus-954.16-Cc_AM_T3-SIntR1rc    TGCCTGTAATCCCAGCTACTCAGGAGG
Sapajus-954.16-Cc_AM_T3-R
Sapajus-954.16-Cc_AM_T3-IntF1     CNTGCCTGTAATCCCAGCTACTCAGGAGGCTGAGGCAGGAGAATTGCCTGAACCCAGGAGGCGGAGGTTGCGGTGAGCCGAGATCGCGCCATTGCACTCC
Sapajus-954.16-Cc_AM_T3-F_rc      ~~~~~~~~~~~~~~~~~~~~~~~~~~~~~~~~~~~~~~~~~~~~~~~~~~~~~~~~~~~~~~~~~~~~~~~~~~~~~~~~~~~~~~~~~~~~~~~~~~~~
Sapajus-954.16-UF31995-SIntR1rc     TGCCTGTAATCCCAGCTACTCAGGAGGCTGAGGC
Sapajus-954.16-UF31995-R
Sapajus-954.16-UF31995-F_rc       ~~~~~~~~~~~~~~~~~~~~~~~~~~~~~~~~~~~~~~~~~~~~~~~~~~~~~~~~~~~~~~~~~~~~~~~~~~~~~~~~~~~~~~~~~~~~~~~~~~~~

                                           710       720       730       740       750       760       770       780       790       800
                                  ....|....|....|....|....|....|....|....|....|....|....|....|....|....|....|....|....|....|....|
Reverse Complement
Sapajus-954.16-S. apella genome   GCCCCTAGGGttgacagaaggggaaaaaaagagaaaacaaatgagagTGTGGTTTAGAAAGTAATATTCGTAAAATGAACTTAGAGtggtacaataataa
Sapajus-954.16-C. imitator geno   ~~~~~~~~~~~~~~~~~~~~~~~~~~~~~~~~~~~~~~~~~~~~~~~~~~~~~~~~~~~~~~~~~~~~~~~AAAATGAACTTAGAGTGGTACAATAATAA
Sapajus-954.16-Cc_AM_T3-SIntR1rc
Sapajus-954.16-Cc_AM_T3-R
Sapajus-954.16-Cc_AM_T3-IntF1     AGCCTGGGTAACATGAGTGAAACTCCGTCTCAAAAAAA
Sapajus-954.16-Cc_AM_T3-F_rc      ~~~~~~~~~~~~~~~~~~~~~~~~~~~~~~~~AAAAAAAAAAAAAAAAAAAAAAAAGAAAGTAATATTCATAAAATGAACTTAGAGTGGTACAATAATAA
Sapajus-954.16-UF31995-SIntR1rc
Sapajus-954.16-UF31995-R
Sapajus-954.16-UF31995-F_rc       ~~~~~~~~~~~~~~~~~~~~~~~~~~~~~~~AAAAAAAAAAAAAAAAAAAAAAAAAGAAAGTAATATTCATAAAATGAACTTAGAGTGGTACAATAATAA
```
